# Increased Nur77 is disconnected from TCR affinity in insulin-specific Tregs

**DOI:** 10.1093/jimmun/vkag136

**Published:** 2026-07-02

**Authors:** Yi Jing, Yuelin Kong, Baoyu Liu, Elizabeth Kolawole, Tessa Galland, Luke G Gillen, Maren Kenison, Maran Sprouse, Brian D Evavold, Matthew L Bettini, Maria Bettini

**Affiliations:** Department of Pathology, Division of Microbiology and Immunology, University of Utah, Salt Lake City, UT, United States; Section of Diabetes and Endocrinology, Department of Pediatrics, Texas Children’s Hospital, Baylor College of Medicine, Houston, TX, United States; Section of Diabetes and Endocrinology, Department of Pediatrics, Texas Children’s Hospital, Baylor College of Medicine, Houston, TX, United States; Department of Pathology, Division of Microbiology and Immunology, University of Utah, Salt Lake City, UT, United States; Department of Pathology, Division of Microbiology and Immunology, University of Utah, Salt Lake City, UT, United States; Department of Pathology, Division of Microbiology and Immunology, University of Utah, Salt Lake City, UT, United States; Department of Pathology, Division of Microbiology and Immunology, University of Utah, Salt Lake City, UT, United States; Department of Pathology, Division of Microbiology and Immunology, University of Utah, Salt Lake City, UT, United States; Section of Diabetes and Endocrinology, Department of Pediatrics, Texas Children’s Hospital, Baylor College of Medicine, Houston, TX, United States; Department of Pathology, Division of Microbiology and Immunology, University of Utah, Salt Lake City, UT, United States; Department of Pathology, Division of Microbiology and Immunology, University of Utah, Salt Lake City, UT, United States; Department of Pathology, Division of Microbiology and Immunology, University of Utah, Salt Lake City, UT, United States

**Keywords:** T cells, autoimmunity, T cell receptors, type 1 diabetes, regulatory T cells, TCR repertoire

## Abstract

Foxp3+ regulatory T cells (Tregs) are capable suppressors of aberrant self-reactivity. However, how differences in affinity and specificity may support Treg function compared with autoimmune T cell function remains unresolved. In this study, we analyzed the T cell receptor (TCR) repertoires of the regulatory and effector T cells that spontaneously infiltrate pancreatic islets of nonobese diabetic mice and therefore share antigen specificity. Using 2-dimensional micropipette measurements of TCR affinity, we show that effector and regulatory T cell-derived TCRs possess similar wide-ranging affinities for self-antigen. Treg-derived TCRs conferred variable protective function and Treg suppressive capacity was, in part, determined by the relative antigen-reactivity of effector T cells. Interestingly, when expressing the same TCR, Tregs showed higher Nur77-GFP expression than effector T cells in vivo, suggesting a Treg-intrinsic ability to compete for antigen. In vitro, we observed accelerated Treg TCR activation, suggesting that Tregs are poised for faster response to antigen than T effectors. Our findings expose a subpopulation of Tregs possessing low-affinity, suboptimal TCRs, obscured by apparent higher Nur77 expression in Tregs as a whole.

## Introduction

Type 1 diabetes (T1D) is characterized by autoimmune targeting of beta cells mediated by beta cell antigen-specific T cell responses.^1^ CD4+Foxp3+ regulatory T cells (Tregs) effectively suppress anti-beta cell autoimmunity but ultimately fail in T1D.^2^ The differences in the T cell receptor biophysical parameters that support Treg versus effector T cell function in autoimmunity are not fully resolved. The thymic development of Tregs is directed by T cell receptor (TCR) specificity and affinity and is heavily dependent on the thymic presentation of cognate antigens.^3–6^ As a result, thymically derived Tregs express a diverse TCR repertoire with strong self-reactivity and minimal overlap with CD4+ Foxp3– effector T cells (Teffs).^7–9^ However, the reported lack of TCR overlap and uniqueness of the Treg TCR repertoire is primarily derived from sequencing polyclonal T cells without focusing on a particular antigenic specificity. Indeed, when a single antigen specificity is explored, the level of repertoire overlap between Tregs and Teffs can be as high as 40%.^10^^,^^11^ Moreover, in experimental autoimmune encephalomyelitis model for multiple sclerosis, Treg and Teff TCRs were shown to exhibit similar ranges of affinity and reactivity for myelin oligodendrocyte glycoprotein.^12^ These observations suggest not only that high self-reactivity may fail to guarantee development into a Treg lineage, but also that low affinity for self antigen may be sufficient for Treg development. However, due to the rarity of Treg cells of a single peptide specificity, it remains unclear whether Treg development and recruitment to the tissue in a polyclonal environment can be supported by a wide range of TCR reactivities, and whether all tissue Treg-derived TCRs are functional in protection against autoimmunity.

Tissue specificity is thought to be necessary for optimal Treg efficacy in T1D, but our limited understanding of how the TCR controls Treg function is an obstacle to developing optimal antigen-specific Treg therapies.^13–15^ In our previous studies, we observed that Treg function in the nonobese diabetic (NOD) mouse model correlated positively with self-reactivity defined by stronger TCR signaling (Nur77-GFP) in the tissue, as well as higher levels of CD5, indicating stronger TCR signaling during thymic selection.^16^ However, Tregs expressing insulin-specific low-affinity TCRs were also capable of contributing to the regulation of autoimmune diabetes while exhibiting a distinct transcriptional profile,^16^^,^^17^ indicating that high- and low-affinity Tregs may play nonredundant roles in T1D prevention. Moreover, a recent study comparing Tregs expressing engineered TCRs of varying avidities observed that low avidity TCRs supported the highest functionality.^18^ These observations suggest that Tregs prefer lower-affinity TCRs for certain functions, and that the TCR affinities effectively supporting Treg development might differ from the TCR affinities supporting optimal Treg function.

In this study, we utilized insulin tetramer–specific T cell isolation to compare TCR repertoires of pancreatic islet infiltrating Treg and Teff cells in the context of autoimmune diabetes. Surprisingly, the study revealed a similar breadth of insulin-specific TCR affinities between Tregs and Teffs, despite higher Nur77-GFP and CD5 expression in islet-infiltrating insulin-specific Tregs. Using TCR retrogenic (Rg) mice, adoptive transfer, and TCR-redirected Tregs, we show that Treg-derived TCRs varied in their abilities to confer optimal Treg function. Interestingly, Tregs exhibited a cell-intrinsic capacity to maximize low-affinity TCRs for increased downstream signaling compared with Teffs, suggesting that lower-affinity TCRs could be sufficient for Treg function. However, when paired against higher-affinity effector T cells, Tregs with lower functional affinity were insufficient. In summary, we detected a TCR-intrinsic insufficiency relative to Teffs in tissue-specific low-affinity Tregs that may contribute to the pathogenesis of T1D.

## Methods

### Mice

NOD/ShiLtJ (NOD), NOD/ShiLt-Tg(Foxp3-EGFP/cre)1cJbs/J (NOD.Foxp3-GFP), NOD.Cg-*Prkdc^scid^*/J (NOD.scid), and NOD.*Tcra^tm1Mjo/DoiJ^* (NOD.TCRαKO) mice were purchased from the Jackson Laboratory. B6.Nur77^GFP^ mice^19^ were backcrossed to NOD for at least 10 generations and subsequently crossed to NOD.*scid.*^20^ NOD.TCRαKO.Ins1KO.Ins2KO.InsY16A mice were generated by crossing NOD.Cg-Tg(Ins2*Y16A)3Ell *Ins1^tm1Jja^ Ins2^tm1Jja^*/GseJ mice^21^ with NOD.TCRαKO mice.^6^ NOD.TCRαKO.Foxp3-GFP mice were generated by crossing NOD.Foxp3-GFP mice with NOD.TCRαKO mice. Unless specified, mice of both sexes were used in experiments. All mice were housed under specific pathogen–free conditions in Baylor College of Medicine and University of Utah facilities. All experiments were performed in accordance with Institutional Animal Care and Use Committee protocols at Baylor College of Medicine and the University of Utah.

### Flow cytometry and fluorescence-activated cell sorting

Surface staining of cell lines and primary cells was performed in phosphate-buffered saline (PBS) with 3% fetal bovine serum (FBS) and 0.05% sodium azide. Fc receptor was blocked by incubating with TruStain FcX reagent (BioLegend; 101320). Viable cells were identified using Zombie Dyes (BioLegend; 423109&423107). For intracellular staining, cells were fixed using the eBioscience Foxp3/Transcription Factor Fixation/Permeabilization (00-5521-00) or 2% methanol-free paraformaldehyde (Thermo Fisher Scientific; 28908) at room temperature for 30 min. Fixed cells were washed twice before intracellular staining in eBioscience Permeabilization Buffer (00-8333-56) on ice overnight. Flow cytometry analyses were performed on LSRFortessa II (BD Biosciences) or Aurora (Cytek). Fluorescence-activated cell sorting was performed using BD FACSAria II/III or Sony MA900 cell sorter. Data were analyzed with FlowJo 10 software (TreeStar). Monoclonal antibodies against following markers were used: CD4 (GK1.5), CD8α (53-6.7), CD5 (53-7.3), CD3 (145-2C11), TCRβ (H57-597), IFNγ (XMG1.2), CD62L (MEL-14), CD44 (IM7), CD25 (PC61), GITR (TYGITR 765), CD45.1 (A45), CD45.2 (104), CD11b (M1/70), CD11c (N418), B220 (RA3-6B2), PD-1 (29F.1A12), CD73 (TY/11.8), ICOS (D10.G4.1), and CTLA4 (UC10-4B9) from BioLegend; Nor1(H-7) from Santa Cruz; ki67(B56 BD) and Lag3(C9B7W BD) from BD; Foxp3 (FJK-16s), CD11b (M1/70), B220 (RA3-6B2), γδTCR (UC7-13D5), TER-119 (TER-119), CD49b (DX5), and Nur77(12.14) from eBioscience; and TCR Vβ4 (REA729) from Miltenyi Biotec. InsB:9–23 E21G/R22E tetramer (Ins-tet) was obtained from the National Institutes of Health Tetramer Core Facility. Tetramer staining was performed for 1 h on ice.

### Peptide

InsB:9–23 ‘wild-type’ with cysteine substituted for alanine (SHLVEALYLVAGERG), R22E (SHLVEALYLVAGEEG), and E21G, R22E (SHLVEALYLVAGGEG) peptides were purchased from GenScript, >90% pure by high-performance liquid chromatography.

### Generation of TCR Rg mice

TCR Rg mice were generated as previously described.^22^ Briefly, 5-fluorouracil (NDC; 63323-117-51) was given intraperitoneally to bone marrow (BM) donor mice (0.15 mg/g body weight). Donor mice, 6 to 15 wk of age, were sacrificed 3 d later, and isolated BM cells were cultured in Dulbecco’s Modified Eagle Medium with 20% FBS and supplements, including nonessential amino acids (Quality Biological; 116-078-721), glutamine (Corning; 25-005-CI), sodium pyruvate (Corning; 25-000-CI), HEPES (Corning; 25-060-CI), beta-mercaptoethanol (Gibco; 21-985-023) and penicillin-streptomycin) (Corning; 30-002-CI), and cytokines (mSCF, IL-6, hIL-3). Retroviral supernatant was generated using TCR vector–transduced GP+E86 cells. BM cells were transduced with retroviral supernatant twice by spinoculation (2,500 rpm, 1 h, 37 °C). Transduced BM cells (2–4 million) were transferred intravenously into sublethally irradiated (500 rads) 6- to 12-wk-old NOD.TCRαKO mice. Recipient mice were equally distributed among groups based on age and sex. Unless otherwise stated, NOD.*scid* mice were used as BM donors.

### Assessment of diabetes

TCR Rg mice were monitored for diabetes weekly, starting from 5 wk after BM transfer. Diabetes onset was defined as blood glucose ≥400 mg/dL or ≥300 mg/dL for 2 consecutive days.

### Isolation of pancreatic islets

T cells that infiltrate pancreatic islets were isolated after intrabile duct injection and digestion with collagenase IV (Worthington Biochemical).^20^ Perfused pancreata were incubated at 37 °C for 30 min and washed twice with 5% FBS+Hanks’ Buffered Salt Solution (Corning). Islets were hand-picked and dissociated at 37 °C in 1 mL of cell dissociation buffer (Gibco) for 15 min. Dissociated islets were washed with Hanks’ Buffered Salt Solution before proceeding to analysis.

### TCR repertoire sequencing

For TCRβ library preparation for ImmunoSEQ, CD4+ Teffs (Foxp3-GFP negative) and Tregs (Foxp3-GFP positive) isolated from pancreatic islets of P2-TCRα Rg mice were stained with PE and allophycocyanin conjugated insulin Ins-tet. Tetramer-positive cells were sorted into ATL buffer (Qiagen) and stored at −80 °C. Splenocytes of P2-TCRα Rg mice were enriched for CD4+ cells using CD4 microbeads (Miltenyi Biotec, 130-117-043). Teffs (≤140,000 cells per mouse) and Tregs were sorted from enriched cells into ATL buffer and stored at −80 °C without tetramer staining. Genomic DNA was purified from frozen samples using QIAamp DNA Micro Kit (Qiagen). Sequencing data were analyzed using the ImmunoSEQ analyzer (version 3.0) and vegen and vvenn R packages.

### TCR-beta sequence alignment

The CDR3β amino acid sequences of insulin tetramer binding Treg and Teff cells isolated from pancreatic islets of P2-TCRa Rg mice were matched against public TCR databases, namely McPAS-TCR (https://friedmanlab.weizmann.ac.il/McPAS-TCR/),^23^ and Immune Epitope Database (IEDB) (https://www.iedb.org/). If a TCR with identical CDR3β and TRBV gene was identified, it was considered a direct match. CDR3β amino acid sequences were also queried using the IEDB TCRMatch tool (http://tools.iedb.org/tcrmatch/)^24^ with a score threshold of 0.90. Potential antigen specificity was predicted based on sequence similarity to TCRs in the database.

### Functional avidity measurements

TCR functional avidity was measured using TCR transduced 4G4.CD4+ thymoma cells or in vitro expanded Rg CD4+ T cells. TCR negative 4G4.CD4+ cells were transduced with TCR expression vectors by 2 consecutive spinoculations (1,800 rpm, 20 °C, 90 min) performed 1 d apart. Transduced cells were purified by CD3ε+ MACS enrichment. A total of 50,000 TCR+ 4G4.CD4+ cells were stimulated with plate-bound tetramer or peptide and 25,000 I-A^g7^–expressing M12.C3 lymphoma cells. Plates were coated with tetramer for 12 h at 4 °C at 1:1,000 in PBS (10 nM). After 24 h, IL-2 concentration in supernatant was measured by ELISA. For IL-2 ELISA, 96-well high-adhesion plates (VWR; 490012-252) were coated with IL-2 antibody (BioLegend; 503702) and blocked using 1% bovine serum albumin (Fisher Scientific; BP9703-100). Biotinylated IL-2 antibody (eBioscience; 13-7021-85), SA-HRP (BioLegend; 405306), and TMB (BD Biosciences; 555214) were used for detection. Reaction was stopped by adding sulfuric acid and absorbance at 450 nm was then measured. For TCR functional avidity measurement using Rg T cells, spleens of TCR Rg mice were dissected and homogenized, and CD4+ T cells were isolated using MACS enrichment. Purified CD4+ T cells were expanded by stimulating T cells with PMA (Sigma-Aldrich; 10 ng/mL) and ionomycin (Sigma-Aldrich; I0634, 1 µg/mL) for 48 h and followed by expansion with 1,000 U/mL hIL-2 (PeproTech) for 2 wk. Expanded T cells were stimulated with peptide and dendritic cells obtained from B16.Flt3l melanoma immunized NOD.TCRαKO mice.^25^ Cells were stimulated for 5 h in the presence of brefeldin A and monensin (BioLegend). Intracellular IFNγ was measured by flow cytometry.

### 2-Dimensional micropipette affinity measurement

TCR 2-dimensional affinity was measured using the micropipette adhesion frequency assay which was previously described in detail.^26^^,^^27^ Briefly, biotin-tagged peptide–major histocompatibility complex (MHC) monomers were coated on biotinylated human red blood cells (RBCs) via biotin-streptavidin chemistry. Individual coated RBCs were used as antigen-presenting cells and were mechanically controlled to approach, contact (for 2 s), and retract from CD4+ T cells placed on opposing micropipettes. This cycle was performed 50 times per T cell/RBC pair. The presence of adhesion (indicating TCR–pMHC ligation) was read out as elongation of the RBC membrane during retraction and adhesion frequency (Pa) was calculated as the number of adhesion events divided by total cycle number. Cells with Pa greater than the cutoff for nonspecific binding (to CLIP/I-A^g7^, nominally 10%) were deemed as antigen specific. For each cell pair, 2-dimensional affinity (A_c_K_a_) was calculated using the following equation:


$$A_{c}K_{a}=\frac{-\ln(1-\mathrm{Pa}(\infty ))}{m_{R}m_{L}}$$

where Pa(∞) is adhesion frequency at an equilibrium contact time (≥2 s) and where m_R_ and m_L_ are densities of the receptor (TCR) and ligand (pMHC), respectively.

### Treg and Teff cell purification and T cell TCR transduction

CD4+ T cells were isolated from spleens and nonpancreatic draining lymph nodes (ndLNs) by MACS enrichment (Miltenyi Biotec). Treg and Teff cells were then sorted from CD4+ T cells (Teff: CD4+CD5+CD25–GITR^lo^; Treg: CD4+CD5+CD25+GITR^hi^). Postsort purity was determined by intranuclear Foxp3 staining (≥95% for polyclonal primary T cells and Rg Teffs; ≥ 90% for Rg Tregs). For TCR transduction, sorted cells were stimulated with anti-CD3– and anti-CD28–coated dynabeads (Thermo Fisher Scientific; beads:cells = 1:1) and were transduced retrovirally by spinoculation (2,500 rpm, 37 °C, 1 h) at 24 h and 48 h after stimulation. Teffs were supplemented with 50 U/mL hIL-2 (PeproTech) and Tregs with 1,000 U/mL hIL-2.

### In vitro T cell stimulation and kinetics of Nur77 and Nor1 expression

Tregs and Teffs were sorted from NOD mice and plated at 100,000 per well of a 96-well U-bottom plate then rested for 20 h without stimulation. Tregs were maintained with 1,000 U/mL hIL-2, and Teffs with 100 U/mL hIL-2. For stimulation, a flat-bottom 96 well plate was coated for 12 h at 4 °C with 1 µg/mL anti-CD3e in PBS, and the plate was washed 2 times with PBS. A total of 50,000 Tregs or Teffs were deposited into the coated wells. Tregs and Teffs were collected from the plate at various timepoints and analyzed by flow cytometry.

### Statistical analysis

All data analysis was performed using GraphPad Prism 10 (GraphPad Software). Statistical significance between sample means was indicated as such: *P < 0.05; **P < 0.01; ***P < 0.001; ****P ≤ 0.0001.

### Study approval

All mouse experiments were approved and conducted under the auspices of the University of Utah (Institutional Animal Care and Use Committee, protocol 00001509) and Baylor College of Medicine (Institutional Animal Care and Use Committee, protocol AN-6394).

## Results

### Insulin-specific Tregs and Teffs exhibit similar range of TCR affinities

**Figure 1:**
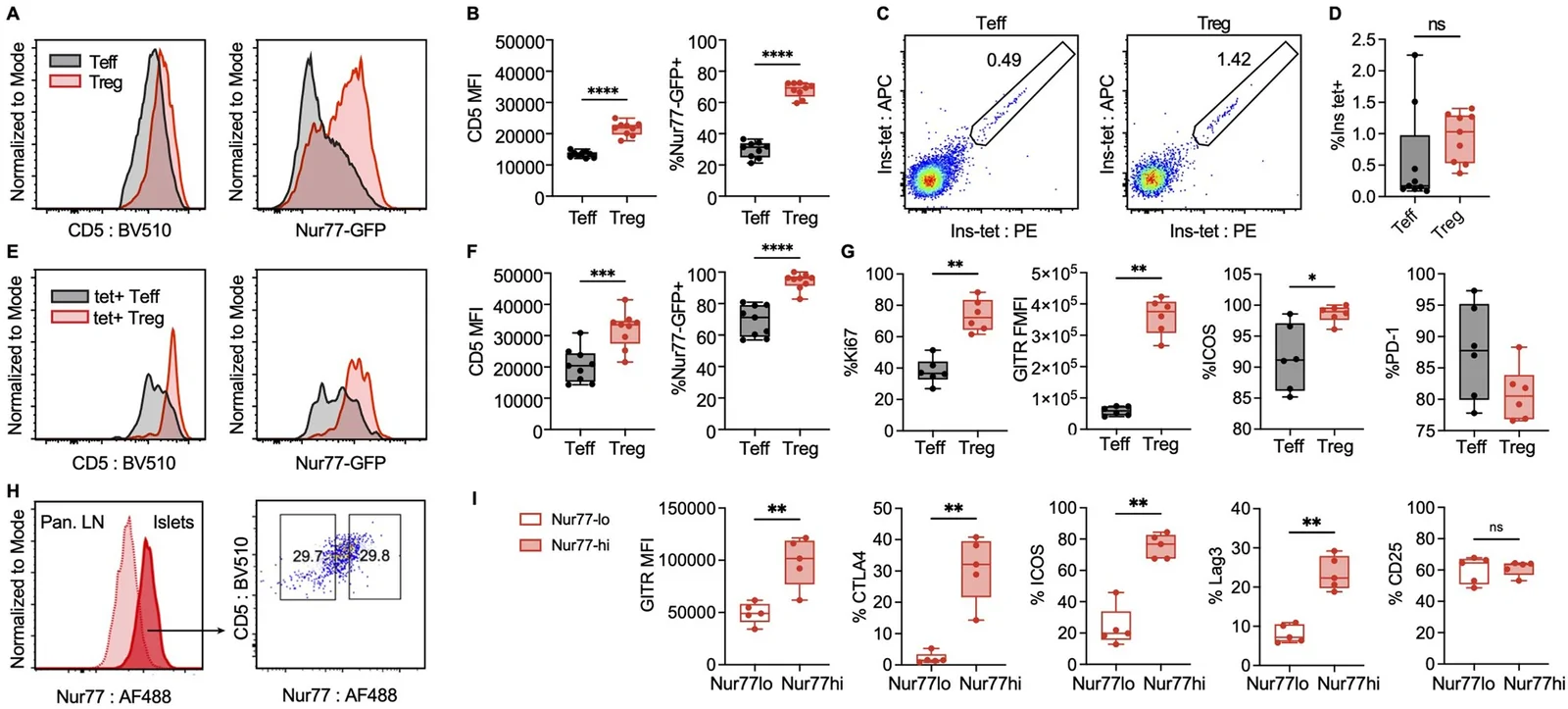
Islet-infiltrating insulin-specific Tregs show a higher level of self-reactivity and TCR signaling than Teffs. Teffs and Tregs were isolated from infiltrated pancreatic islets of prediabetic 10- to 12-wk-old NOD.Nur77-GFP females, stained with InsB:10–23/I-A^g7^ tetramer (Ins-tet), and analyzed by flow cytometry. (A, B) Representative flow cytometry plots and summary of CD5 and Nur77-GFP expression in all islet Tregs and Teffs. (C, D) Representative flow plots and summary of Ins-tet+ frequencies in islet Teffs and Tregs. (E, F) Representative flow plots and summary of CD5 and Nur77-GFP expression levels in islet ins-tet+ Teffs and Tregs. Each dot represents islet T cells from a single mouse (n = 9 mice). (G) Summary of data for Ki67, GITR, ICOS, and PD-1 expression on Ins-tet+ Tregs and Teffs in the pancreatic islets of 12- to 15-wk-old prediabetic NOD.Nur77-GFP female mice (n = 6). Gating strategy in Fig. S1A, E, F. (H, I) T cells from the islets and pancreatic lymph nodes (Pan. LN) of prediabetic 12-wk-old NOD female mice were stained for intracellular Nur77 (H), and analyzed by flow cytometry. (I) Analysis is gated on CD4+CD3+CD5+Foxp3+ followed by top 30% and bottom 30% Nur77 expression. Each dot represents islet T cells from a single mouse. Representative flow plots are in Fig. S1J. Statistical analysis was performed using Mann-Whitney (n = 5). *P ≤ 0.05, **P ≤ 0.01, ***P ≤ 0.001, ****P ≤ 0.0001. *P* > 0.05 was not significant (ns). APC, antigen-presenting cell; MFI, mean fluorescence intensity.

Previous studies have observed higher self-reactivity and stronger TCR signaling in CD4+Foxp3+ Tregs in the periphery and in the pancreatic islets of NOD mice, as evidenced by higher expression levels of CD5 and Nur77 compared with CD4+Foxp3– Teffs.^16^^,^^19^ We confirmed previous observations that CD4+Foxp3+ Tregs in the islets exhibit higher CD5 and Nur77-GFP expression (Fig. 1A, B).^16^^,^^19^ Frequencies of insulin B9-23 tetramer+ (Ins-tet+) cells were generally higher in Tregs compared with Teffs; however, the trend did not reach significance (Fig. 1C, D). After gating on Ins-tet+ cells, Tregs expressed significantly higher levels of CD5 and Nur77-GFP (Fig. 1E, F), indicating that the increased self-reactivity in Tregs was not dependent on different TCR specificities compared with Teffs. To verify that the differences in Nur77-GFP were not affected by the stability of the Nur77-GFP protein,^28^ we directly stained for intracellular Nur77 and Nor1. Nur77 is thought to be primarily regulated by phospho-Erk downstream of TCR activation, while Nor1 is more dependent on calcium flux and NFAT.^28^ Nur77 and Nor1 were both increased in islet Tregs (Fig. S1A–C). When we gated on the same level of Nur77 expression in Teffs and Tregs, we did not observe a difference in Nor1, supporting a direct correlation between Nur77 and Nor1 expression (Fig. S1D).

Nur77 has been associated with anergic and exhausted phenotypes characterized by loss of proliferation and increased expression of inhibitory receptors.^29–31^ Analysis of Ins-tet+ Tregs and Teffs showed higher levels of Ki67, a marker of cell cycle progression, and costimulatory receptors GITR and ICOS in Tregs but no increase in the inhibitory receptor PD-1 (Fig. 1G; Fig. S1E, F).^32^ We then stratified tetramer-negative Nur77-GFP+ Treg and Teff populations based on expression of Nur77 and observed that increasing levels of Nur77 did not inhibit expression of Ki67 but were associated with upregulation of costimulatory receptors GITR and ICOS in Foxp3+ cells (Fig. S1G–I). Expression of inhibitory PD-1 corresponded with an increase in Nur77; however, the levels of PD-1 in Tregs did not surpass those in Teffs (Fig. S1H, I). TCR signaling in Tregs can regulate multiple pathways important for Treg function.^33^ To confirm that Nur77 expression was indeed correlated with activation of genes downstream of TCR signaling, we compared Nur77 high (top 30%) and Nur77 low (bottom 30%) Tregs in the islets of NOD mice (Fig. 1H, I; Fig. S1J). Consistent with previous studies that defined TCR induced gene signature in Tregs,^34^^,^^35^ we observed a positive correlation of Nur77 with GITR, CTLA4, ICOS, and Lag3 (Fig. 1I;Fig. S1J). There was no correlation with CD25, a receptor chain sensitive to the levels of IL-2 but not TCR signaling.^34^ In combination, the data show that Tregs exhibit increased Nur77 and Nor1 that correspond with their increased activation in the tissue.

**Figure 2:**
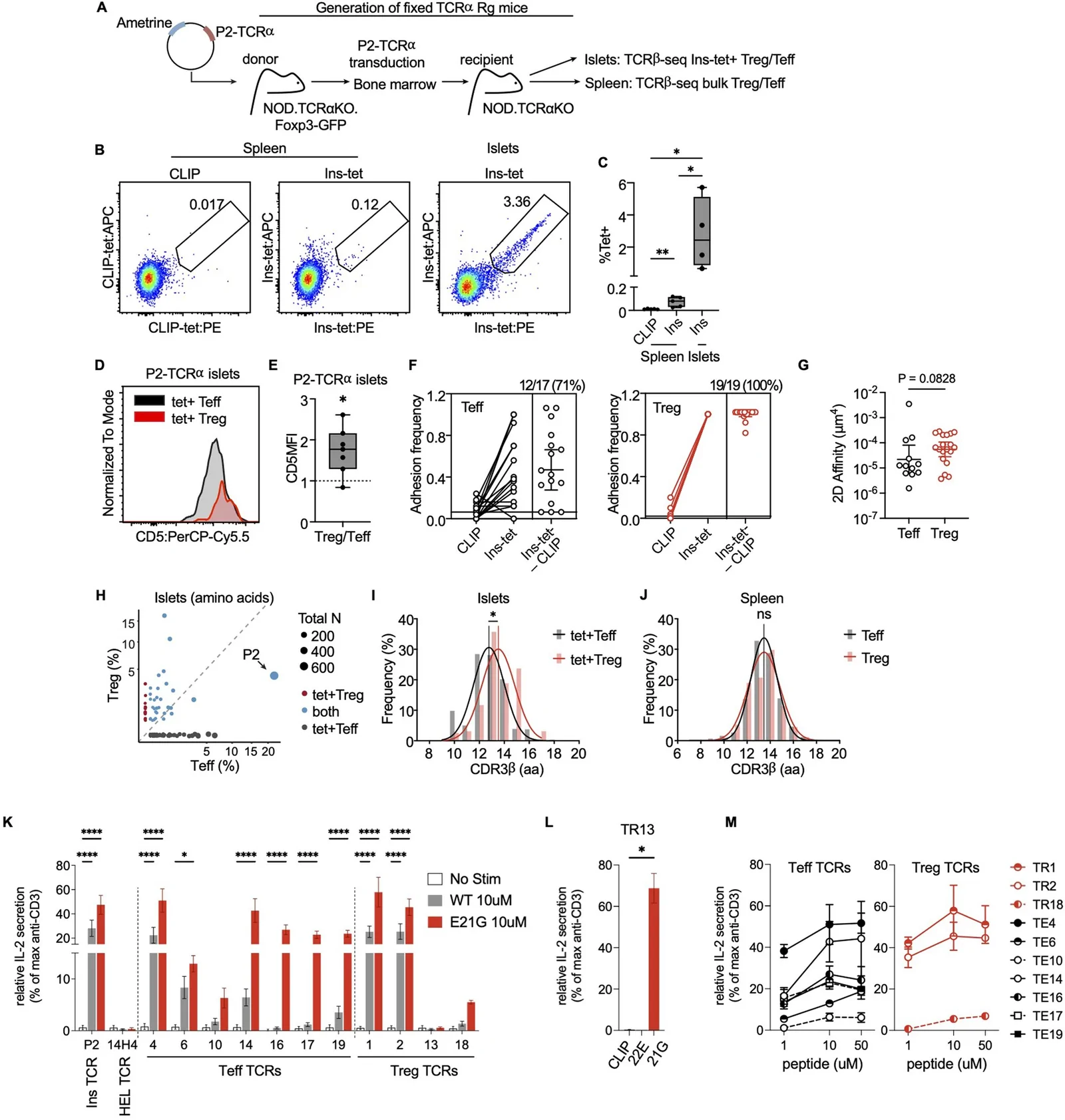
Pancreatic islet-infiltrating tetramer–positive Tregs exhibit a wide range of reactivity to insulin and possess unique TCR characteristics compared with Teffs. BM hematopoietic stem cells collected from NOD.TCRαKO.Foxp3-GFP mice were transduced with a retroviral vector that encoded the TCRα chain of insulin-specific TCR P2 and Ametrine fluorescent reporter. Transduced BM cells were transferred intravenously into irradiated NOD.TCRαKO recipients. Recipients were sacrificed 10 to 12 wk later, and Ametrine+CD4+ T cells were isolated from spleens and pancreatic islets. Splenic GFP+ Tregs and GFP– Teffs were sorted individually. Islet samples from 38 mice were pooled and stained with Ins-tet. Ins-tet+ Teffs and Tregs were sorted. Genomic DNA was purified from sorted samples and TCRβ repertoires were sequenced. (A) Schematic diagram of the experimental design. (B, C) Frequencies of insulin or CLIP tetramer–positive CD4+ T cells in spleens and pancreatic islets of P2-TCRα Rg mice. Welch *t* test followed by Bonferroni correction (n = 4 or 5). (D, E) CD5 expression in Ins-tet+ Tregs and Teffs isolated from pancreatic islets of P2-TCRα mice. Data are pooled from 3 independent experiments (n = 7) and analyzed by 1-sample *t* test. (F, G) Eight prediabetic P2-TCRα females were sacrificed 14 wk after BM transfer. Teffs and Tregs were sorted from pancreatic islets. (F) Tetramer binding frequencies and (G) 2-dimensional (2D) affinity of insulin-specific cells were measured using the 2D-MP adhesion frequency assay against InsB:10–23/I-A^g7^. Insulin-specific cells were defined as having higher binding frequency to insulin over CLIP/I-A^g7^. Numbers and percentage of insulin-specific cells identified of total Tregs and Teffs measured were shown. Data are plotted as geometric means ± 95% confidence interval and analyzed by Mann-Whitney test. (H) TCR repertoire (amino acid) overlap between islet ins-tet+ Tregs and Teffs. Dot size represents numbers of cells included in a specific clonotype. The dominant T cell (Continued).clone that expressed the same TCRβ as the parent TCR P2 is indicated with an arrow. (I, J) Distributions of CDR3β length in (I) islet Ins-tet+ and (J) splenic repertoires. Extra sum-of-squares *F* test. *P ≤ 0.05. *P* > 0.05 was not significant (ns). (K–M) Ins-tet–binding TCRs identified from pancreatic islets of P2-TCRα Rg mice were expressed in 4G4 thymoma cells. TCR+ 4G4 cells were stimulated with (K) 10 µM of wild-type InsB:9–23 peptide or InsB:10–23_E21G, R22E_ agonist mimotope and M12.C3.g7 cells, (L) plate-bound insulin or control CLIP tetramers, or (M) varying concentrations of B:10–23_E21G, R22E_ agonist mimotope and M12.C3.g7 cells for 24 h. IL-2 concentrations in the supernatant were measured by ELISA and normalized to anti-CD3 positive control. Data are plotted as means ± SEM and are (K) pooled from or (L) representative of 3 independent experiments and analyzed by Welch analysis of variance followed by Benjamini, Krieger, and Yekutieli’s test. All comparisons are made with negative control. *P ≤ 0.05, **P ≤ 0.01, ****P ≤ 0.0001. *P* > 0.05 was ns. APC, antigen-presenting cell; MFI, mean fluorescence intensity.

TCR signal strength during thymic selection (CD5) and TCR signaling in the tissue (Nur77-GFP reporter) indicated a higher level of self-reactivity of islet antigen-specific Tregs compared with effector CD4+ T cells in the context of tissue-specific autoimmunity and shared antigen. Therefore, we expected islet-infiltrating Treg-derived TCRs to exhibit higher sensitivity and affinity to islet antigens. To compare TCR repertoires of insulin-specific Tregs and Teffs, we generated TCR Rg mice that expressed a fixed TCR alpha chain cloned from a high-affinity InsB:9–23–reactive TCR, P2 (P2-TCRα mice), which paired with an endogenous polyclonal TCRβ repertoire in TCRαKO mice (Fig. 2A).^20^ This approach was necessary to increase the frequency of Ins-tet+ cells amenable for sorting and TCR analysis (Fig. 1C, D; Fig. S2A, B). Although Ins-tet–binding cells were relatively rare in the spleens of P2-TCRα mice, their frequency was significantly increased in the pancreatic islets, enabling downstream analysis (Fig. 2B, C). Islet Ins-tet+ Tregs and Teffs were compared as in Fig. 1, confirming a significantly higher level of CD5 expression on tetramer+ Tregs (Fig. 2D, E). To quantify and analyze affinities of both tetramer+ and low-affinity tetramer– T cells, we applied a 2-dimensional micropipette (2D-MP) adhesion assay that provides physiologically relevant quantification of receptor/ligand interactions embedded in cell membranes.^36^^,^^37^ We sorted Teffs (Foxp3-GFP–) and Tregs (Foxp3-GFP+) from pancreatic islets of P2-TCRα mice and tested T cell binding to InsB:10–23/I-A^g7^ peptide:MHC complexes presented on human RBCs attached to a micropipette. Each T cell was tested 50 times against both insulin and CLIP/I-A^g7^ control RBCs. Positive response to insulin was determined based on binding frequency above the CLIP control, which was set at 10% threshold. Based on positive adhesion frequencies, 71% Teffs and 100% Tregs were determined to be insulin specific (Fig. 2F), suggesting that the majority of Ins-tet–negative islet-infiltrating T cells in P2-TCRα mice were also insulin specific, likely with affinities below the tetramer binding threshold. Effective 2D-MP affinities for the insulin epitope were then calculated for positive binders and showed no significant difference between Tregs and Teffs (Fig. 2G). While the 2D-MP–derived affinities trended higher in Tregs, TCRs in both populations showed a range of affinities spanning at least 100-fold.

To compare TCRβ repertoires between insulin reactive Teffs and Tregs while ensuring antigenic specificity, we focused on tetramer+ T cells isolated from the pancreatic islets of Foxp3-GFP P2-TCRα mice. TCRβ sequences were obtained from 3837 Ins-tet+ Teffs and 499 Tregs isolated from combined islets of 38 P2-TCRα mice. TCRβ repertoires of splenic Tregs and Teffs of 5 individual mice were sequenced without pooling or tetramer staining and were used as a reference library. A total of 358 unique Teff clonotypes and 92 unique Treg clonotypes at amino acid level were obtained from the islets (Table S1), in which 35 were shared between Tregs and Teffs (Fig. 2H;Table S1). Interestingly, this relatively high level of repertoire similarity was also observed in the spleens (Table S1), suggesting an effect of the restricted TCR repertoire rather than iTreg conversion upon entry into the tissue. TRBV13-02 usage was identified most frequently for both Tregs and Teffs, followed by TRBV02-01 and TRBV31-01. The preference for TRBV13-02 was also seen in the spleen and could be explained by effective pairing with the alpha chain of the P2 TCR that was originally paired with TRBV13-02. However, the preference for TRBV02-01 and TRBV31-01 was limited to islets and suggested enrichment based on tissue specificity (Fig. S2C, D). Although we observed similarities in affinities and TRBV usage between Treg and Teff insulin-specific TCR repertoires, islet Ins-tet+ Tregs tended to express longer CDR3β compared with Teffs, which was not observed in splenic repertoires, suggesting Treg preference for structurally different TCRs in the islets (Fig. 2I, J).

### Thymic antigen encounter imprints on islet TCR repertoires

To test the degree to which insulin-specific Treg TCR repertoire characteristics described previously were dependent on the expression of insulin during T cell development, we disrupted T cell recognition by utilizing NOD.Ins1KO.Ins2KO.InsY16A.TCRαKO (NOD.Ins^Y16A^.TCRαKO) mice, which express a form of insulin that is mutated at the key TCR contact residue (Y16A) in the InsB:9–23 epitope.^6^^,^^21^ As diagramed in Fig. S3A, we generated P2-TCRα Rg mice using NOD.TCRαKO mice as BM donors and NOD.Ins^Y16A^.TCRαKO as BM recipients (P2-TCRα.Y16A mice). After development on the Y16A insulin mutant background, CD4+ T cells were isolated from spleens of P2-TCRα.Y16A mice and transferred into new NOD.TCRαKO recipients to allow T cell recognition of insulin and subsequent recruitment to the pancreas. Compared with P2-TCRα mice generated under conditions of normal insulin expression, a significant decrease in islet Ins-tet+ Tregs was observed in mice that received P2-TCRα.Y16A–derived splenic CD4+ T cells, suggesting that peripheral conversion, if any, was not sufficient to compensate for the reduced thymically derived Treg development (Fig. S3B, C). Islet samples were collected from 25 NOD.TCRαKO CD4+ T cell recipients generated from 7 P2-TCRα.Y16A donors, pooled, and sorted based on Ins-tet binding, as was done in Fig. 2. A total of 9,544 Teff and 146 Treg insulin-reactive TCRβ chains were obtained from sequencing, which included 210 unique Teff and 35 Treg clonotypes (Table S1). Ten clonotypes were shared between Teffs and Tregs (4.1% of all clonotypes, 28.6% of Tregs) (Fig. S3D, Table S1). The preference for longer CDR3βs was no longer observed in Tregs (Fig. S3E). Instead, both Tregs and Teffs strongly favored CDR3βs with 13 amino acids. TRBV13-02, TRBV02-01, and TRBV31-01 remained the 3 most dominant TRBV genes identified in Teffs; however, TRBV02-01 and TRBV31-01 lost their dominance in Tregs (Fig. S3F), supporting a role for thymic insulin in shaping Treg TCR repertoire. Together, our data demonstrate that antigen encounter during development and TCR recognition of self-antigen in the tissue leave nonredundant imprints on Treg TCR repertoires.

### Treg TCRs selected from P2-TCRα islet repertoire exhibit a broad range of insulin reactivity

To compare the functional parameters of Treg and Teff TCRs restricted by the same epitope, we selected 18 TCRs from the islet Ins-tet+ repertoires of P2-TCRα Rg mice (Table S2), among which 9 were expressed exclusively by Teff cells (henceforth called Teff or TE TCRs) and 9 were expressed by islet Tregs (Treg or TR TCRs). TCR transfectant cell lines were stimulated with insulin peptides and M12.C3.g7 as antigen-presenting cells. IL-2 secretion was used as a readout of TCR reactivity and normalized against anti-CD3 positive control to eliminate variation due to differences in TCR expression level (Fig. 2K). TCRs without significant response to insulin peptides were also stimulated with plate-bound tetramers to increase sensitivity, which confirmed insulin specificity for TCR TR13 (Fig. 2L). Four out of 9 Treg TCRs and 7 of 9 Teff TCRs were confirmed to be insulin specific (Fig. 2K, L). Query of islet TCR CDR3β sequences in the IEDB public database showed that 29% of Teff and 35% of Treg TCRs were predicted to be insulin specific (Tables S3–S5), including 8 out of the 18 selected TCRs in Table S2. Given that the majority of islet T cells in P2-TCRα mice were determined to be insulin specific using the 2D-MP adhesion assay and IEDB TCRMatch prediction tool (Fig. 2F; Tables S2–S5), nonresponder TCRs in Fig. 2K and 2L likely had functional avidity below the threshold of detection. Among responder TCRs, both the highest and the lowest reactivity were Treg TCRs (1, 2 and 13, 18), suggesting a broad range of reactivity. Importantly, Ins-tet+ Treg TCRs did not show an overall higher reactivity over Teff TCRs, even when tested over a range of peptide concentrations (Fig. 2M). Overall, our observations suggest that a proportion of insulin-specific Tregs recruited to the tissue during autoimmunity express low-affinity TCRs.

### Treg-derived insulin-specific TCRs do not increase Treg development

**Figure 3:**
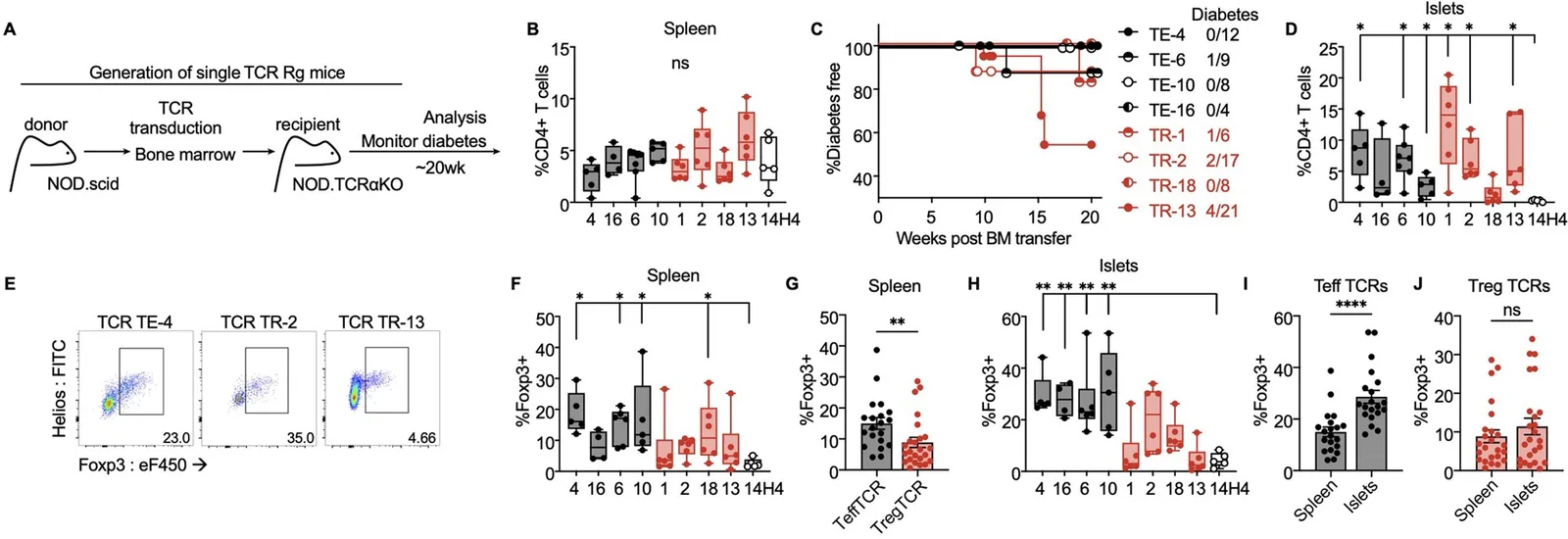
Diabetes and Treg development in Treg and Teff TCR–expressing Rg mice. (A) Rg mice expressing Teff (black), Treg (red) derived TCRs, or hen egg lysozyme–specific 14H4 control TCR (white) were generated by transferring transduced NOD.*scid* BM into irradiated NOD.TCRαKO recipients. Recipient mice were monitored for diabetes and sacrificed for analysis at diabetes onset or 20 wk after BM transfer. (B) Frequencies of CD4+ T cells in spleens. (C) Diabetes incidence. Numbers of mice that developed diabetes out of total shown on the right. (D) Frequencies of CD4+ T cells in pancreatic islets. (E) Representative flow plots of islet-infiltrating T cells from TCR Rg mice gated on CD4+CD5+CD3+. (F) Frequencies of Foxp3+ Tregs among CD4+ T cells in spleens. (G) Splenic Treg frequencies with Treg and Teff TCRs analyzed collectively. (H) Frequencies of Tregs in pancreatic islets. (I, J) Treg frequencies in spleen versus islets of TCR Rg mice expressing (I) Teff-derived or (J) Treg-derived TCRs. Data were pooled from 2 independent experiments. *P ≤ 0.05, **P ≤ 0.01, ****P ≤ 0.0001. *P* > 0.05 was not significant (ns). (B, D, F, H) were analyzed by one-way ANOVA analysis of variance followed by Benjamini-Krieger-Yekutieli multiple-comparisons test (all comparisons made with 14H4 negative control) and (G, I, J) were analyzed using Mann-Whitney test.

We then considered the possibility that Treg-derived TCRs are cross-reactive to other self-antigens or recognize a modified version of the insulin epitope unique to the in vivo environment. To compare the in vivo function of Treg and Teff TCRs, we re-expressed insulin-responsive TCRs selected from Fig. 2 and the hen egg lysozyme–specific control TCR 14H4 in TCR Rg mice. Individual TCRs were expressed by transducing NOD*.scid* BM that was then transferred into NOD.TCRαKO recipients (Fig. 3A). T cell development, recruitment to the tissue, and spontaneous development of diabetes was monitored in TCR Rg mice. No significant difference was observed in splenic CD4+ T cell frequency or number (Fig. 3B; Fig. S4A), indicating comparable peripheral engraftment and T cell development. Spontaneous diabetes development was observed in mice expressing both Treg (TR-1, TR-2, TR-13) and Teff (TE-6) TCRs, and both high-reactivity (TR-1, TR-2) and low-reactivity (TR-13) TCRs (Fig. 3C). All mice except for those expressing TE-16 and TR-18 showed significant islet infiltration compared with mice expressing the negative control TCR 14H4 (Fig. 3D; Fig. S4B). These observations are consistent with our previous findings that TCR reactivity to islet antigen by itself does not predict TCR ability to cause spontaneous diabetes.^20^ They further demostrate that Treg TCRs are not uniquely pathogenic due to higher in vivo antigen sensing or cross-reactivity.

Interestingly, robust Treg development was observed in mice expressing Treg TCRs as well as Teff TCRs (Fig. 3E, F). In fact, when TCRs were analyzed collectively, splenic Treg frequency was significantly higher in Teff TCR Rg mice (Fig. 3G). Importantly, while TR-1 and TR-2 had the highest reactivity to insulin (Fig. 2K), they failed to support better Treg generation than either Treg or Teff TCRs with lower insulin reactivity (Fig. 3F). To exclude the possibility of Treg accumulation over time, we compared splenic Treg frequencies of TCR Rg mice at 2 different time points (10 and 20 wk) after BM transfer and observed no significant difference (Fig. S4C). It has been reported that thymic Treg development is niche sensitive and is limited to a small clonal size.^38^^,^^39^ To determine whether the lower Treg generation in Treg TCR Rg mice was a result of intraclonal competition in the thymus, we cotransferred TCR transduced NOD.*scid* BM with NOD.CD45.2 congenic wild-type BM (Fig. S4D). No significant increase in Treg frequencies or negative correlation between percentages of transduced CD4SP thymocytes and Treg frequencies was observed (Fig. S4E, F) The lower frequencies of Tregs in spleens of Treg-TCR Rg mice were reflected in the pancreatic islets (Fig. 3H). Moreover, while in Teff TCR Rg mice frequencies of Tregs were increased in the islets compared with spleens, such an increase was rarely observed with Treg TCRs (Fig. 3I, J). This suggested that in addition to the reduced Treg development, insulin-specific Treg TCRs might also be inferior in terms of imparting functional fitness to Tregs.

### Low-reactivity TCR-Tregs show impaired function upon adoptive transfer

**Figure 4:**
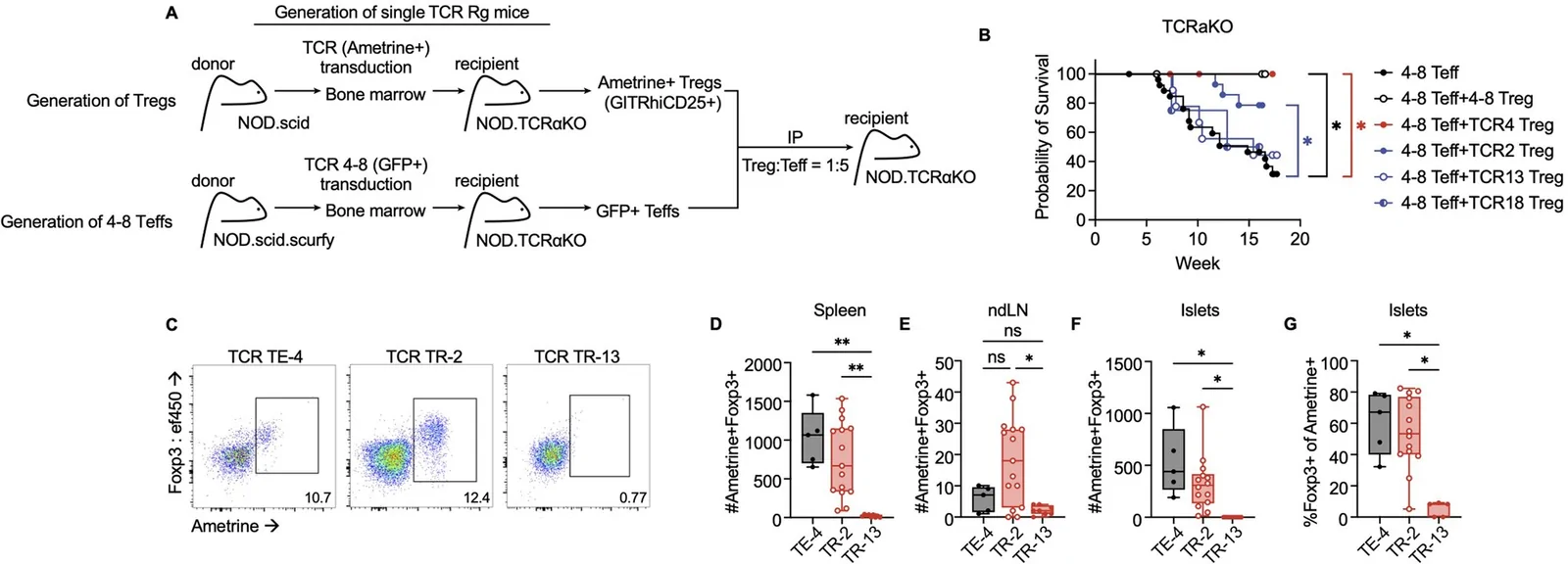
Treg TCR with low-reactivity insulin-specific TCRs show reduced accumulation and protective function in the pancreatic islets. (A) Ametrine+CD4+ Tregs (Ametrine+GITR^hi^CD25+) were sorted from spleens and nonpancreatic islet draining lymph nodes of TCR Rg mice generated using NOD.*scid* BM. The purity of sorted Tregs was confirmed by Foxp3 staining and was consistently >90%. The 4-8 TCR Rg mice were generated using NOD*.scid.scurfy* BM, and CD4+ Teffs cells (GFP+) were sorted from spleens of 4-8 Rg mice and transferred (intraperitoneally [IP]) alone or with Ametrine+Tregs into NOD.TCRαKO recipients (Teff:Treg = 5:1). Recipients were monitored for diabetes for up to 16 wk. (B) Diabetes incidence: 4-8 Teff alone (n = 27); 4-8 Teff+TE-4 Treg (n = 8); 4-8 Teff+TR-2 Treg (n = 14); 4-8 Teff+TR-13 Treg (n = 9). *P ≤ 0.05. *P* > 0.05 was not significant by log-rank test. (C) Representative flow plots of islet-infiltrating T cells. (D–F) Numbers of donor Tregs in spleens, ndLNs, and pancreatic islets of recipient mice. (G) Percentages of Foxp3+ cells within Ametrine+ donor CD4+ T cells recovered from pancreatic islets. (D–G) Kruskal-Wallis test followed by Dunn’s test. *P ≤ 0.05, **P ≤ 0.01. *P* > 0.05 was not significant. Populations with <10 cells and outliers were excluded from analysis using the ROUT method (Q = 1).

To compare the function of Tregs expressing different TCRs in vivo, we tested Treg function against a single pathogenic effector population. Tregs were isolated from pooled spleens and ndLNs of TCR Rg mice. The purity of isolated Rg Tregs were confirmed by ex vivo Foxp3 staining to be >90%. Isolated Tregs were transferred into NOD.TCRαKO female recipients together with Teffs that expressed a highly pathogenic insulin-specific TCR (4-8) (Fig. 4A).^20^ Teff TCR (TE-4), high reactivity Treg TCR (TR-2) as well as control TCR 4-8–expressing Tregs showed effective regulation of 4-8 Teffs and protection against diabetes. However, TR-18 and TR-13 Treg TCRs on the lower end of the reactivity spectrum showed no protection (Fig. 4B). We then used Ametrine fluorescence marker to analyze location and phenotype ofTregs for select high and low-reactivity TCRs. Significantly lower numbers of Foxp3+Ametrine+ (Treg donor) cells were recovered from spleens and islets of TR-13 Treg recipients (Fig. 4C–F), suggesting that TR-13 Tregs had impaired survival capacity and were unable to accumulate in pancreatic islets. Moreover, TR-13 Tregs lost Foxp3 expression, indicating Treg lineage instability (Fig. 4G). Interestingly, when TE-4, TR-2, and TR-13 Foxp3– Teff cells were transferred alone (Fig. S5A), comparable numbers of donor Teffs were recovered from spleen and ndLNs across all TCRs (Fig. S5B). The impaired survival observed in TR-13 with low insulin reactivity appeared to be Treg specific, suggesting that Tregs have more stringent TCR requirements for survival and function.

**Figure 5:**
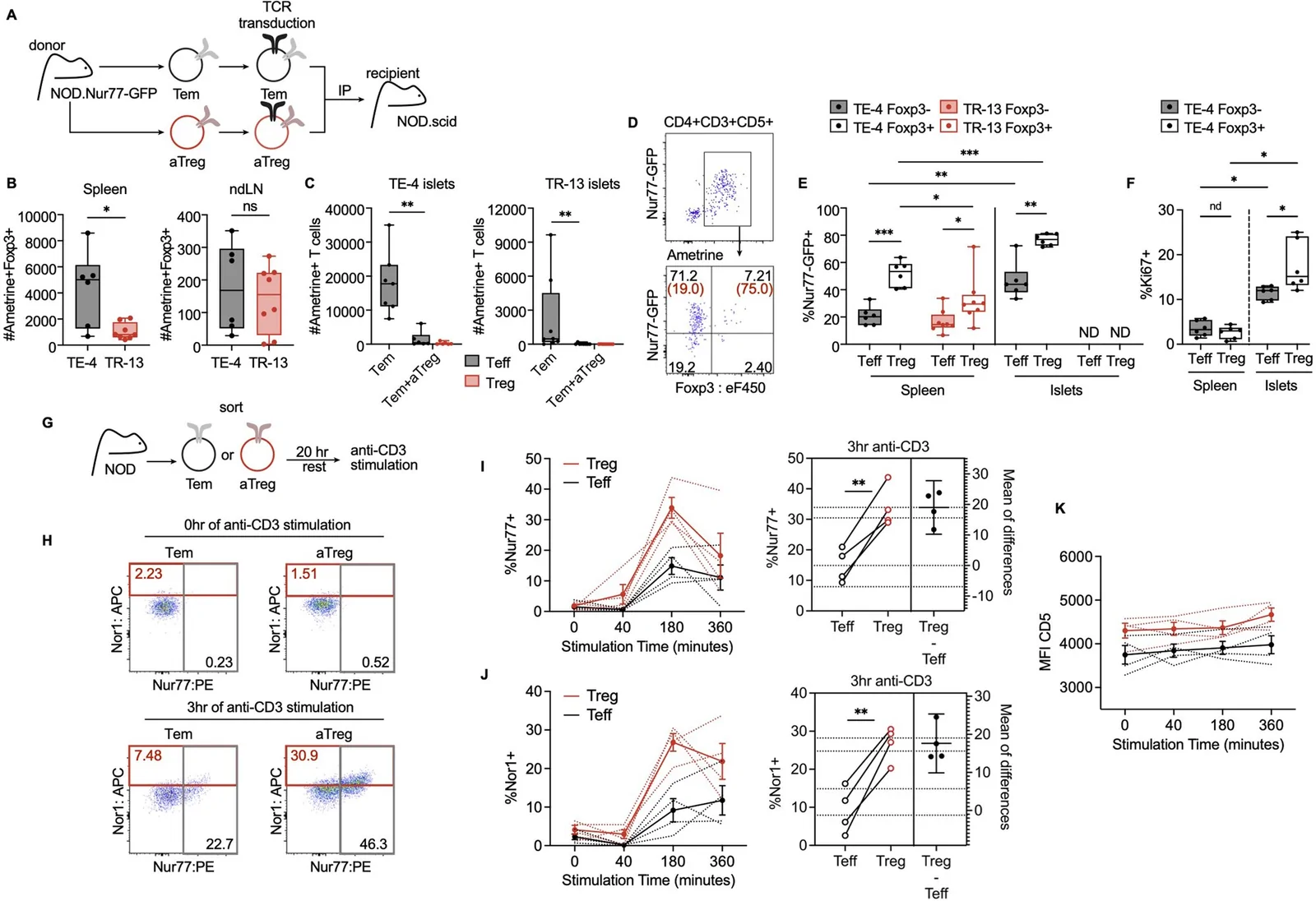
Treg TCR with low insulin reactivity inhibits matching-TCR Teff infiltration into pancreatic islets and exhibits higher TCR signaling compared with effector T cells. (A) aTregs and Tems were sorted from spleens and ndLNs of NOD.Nur77-GFP mice and transduced with indicated TCRs. Transduced (Ametrine+) cells were sorted and transferred (intraperitoneally [IP]) into NOD.*scid* female recipients. Recipients were sacrificed for analysis 2 wk after transfer. Data were pooled from 2 independent experiments. (B) Numbers of Ametrine+ Tregs recovered from spleen and ndLNs. (C) Numbers of Ametrine+ donor T cells in pancreatic islets. (D) Representative flow plots for Nur77-GFP expression in Ametrine-TCR–transduced Tregs and Teffs. (E) Nur77-GFP expression in spleen and islets. (F) Ki67 expression in TE-4 TCR–expressing Tregs and Teffs. Populations with <10 cells and outliers were excluded from analysis using the ROUT method (Q = 1). (G) Schematic of experimental set up. (H, I) aTregs and Teffs were sorted from spleens and ndLNs of NOD mice, rested in vitro for 20 h, and then stimulated with plate bound anti-CD3. Nur77, Nor1, and CD5 expression in cells was measured using flow cytometry. Nor1, Nur77, and CD5 in individual experiments (dotted lines) and as a mean of 4 experiments (solid lines). Data obtained from 4 independent experiments (n = 4). (B, C) Mann-Whitney test; (E, F) Welch analysis of variance followed by Benjamini, Krieger, and Yekutieli’s test; (I, J) paired 2-tailed *t* test.*P ≤ 0.05, **P ≤ 0.01, ***P ≤ 0.001. *P* > 0.05 was not significant (ns). APC, antigen-presenting cell; MFI, mean fluorescence intensity; nd, not detectable.

To exclude the impact of insulin TCRs on Treg development and compare T cell populations with similar levels of activation, we transduced primary polyclonal activated Tregs (aTregs) (CD4+ CD44+CD62L–CD25+GITRhi) and T effector memory cells (Tems) (CD4+ CD44+CD62L–CD25–GITRlo) isolated from spleens and ndLNs of NOD.Nur77-GFP mice with an insulin-specific TCR and cotransferred transduced cells into NOD.*scid* female recipients (Fig. 5A). Consistent with TCR Rg Treg transfers, significantly lower numbers of TR-13 Tregs were recovered from spleens of recipient mice (Fig. 5B), indicating that insulin-reactive Treg survival or expansion is continuously regulated by TCR signaling. To address whether low-reactivity Treg TCRs were sufficient to impart suppressive capacity onto fully mature and functional polyclonal Tregs, we tested the ability of TCR transduced Tregs to suppress Teffs in vivo. Both high- and low-reactivity TCR-transduced aTregs (TE-4 and TR-13 TCRs) inhibited islet infiltration of Tems that expressed the same TCR (Fig. 5C). These observations show that Treg functional capacity varies depending on relative responsiveness of the target T cells. In other words, low-reactivity TR-13 Tregs could suppress TR-13 effectors but were insufficient against highly potent 4-8 effector T cells. An increase in Nur77-GFP expression from spleen to islets was observed in TE-4 Tems and TE-4 aTregs (Fig. 5D, E), validating antigen-specific induction of transduced-TCR signaling. Intriguingly, significantly higher level of Nur77-GFP expression was observed in Tregs compared with Teffs that expressed the same TCR (Fig. 5D, E). In the pancreatic islets, TE-4 TCR–expressing Tregs showed higher levels of Ki67, indicating their increased activation (Fig. 5F). Elevated Treg TCR signal suggested that Tregs are potentially better or faster at responding to antigen in vivo to effectively suppress Teffs with the same TCR.

### Tregs possess a TCR-independent increased antigen recognition capacity compared with Teffs that express the same TCR

To compare function and kinetics of TCR activation in Teffs and Tregs, we sorted polyclonal aTregs (CD44+CD62L–CD25+GITRhi) and Tems (CD62L-CD44+CD62L–CD25–GITRlo) from spleens and ndLNs from young (7–12 wk old) NOD mice. Sorted Tems and aTregs were rested in vitro for 20 h prior to stimulation with plate-bound anti-CD3 (Fig. 5G). Tregs showed accelerated kinetics of Nur77 and Nor1 upregulation in response to antigen stimulation compared with Tems (Fig. 5H–J). As expected, levels of CD5 were maintained at higher levels in Tregs and were not significantly changed in response to TCR stimulation (Fig. 5K). Overall, our data suggest that in addition to the TCR-intrinsic parameters, such as affinity, Tregs possess cell-intrinsic mechanisms to boost their antigen recognition capacity, as measured by activation of downstream gene targets Nur77 and Nor1. The ability of Tregs to increase their sensitivity for antigen might be critically important for their recruitment into the tissue and provide them with a competitive advantage against effector T cells expressing similar TCRs in the context of low antigen availability.

## Discussion

The 2-step instructive model suggests that a strong, transient TCR signal is favored for Treg development.^40–42^ This model is supported by the finding that Tregs express TCRs with stronger self-reactivity than Teff cells. Ablation of cognate antigen expression in the thymus inhibited Treg, but not conventional T cell development, suggesting that the difference in TCR repertoire between the 2 populations is primarily driven by agonist selection of Tregs.^43^ Meanwhile, in a fixed pMHC antigen model ubiquitous expression of antigen resulted in the development of Tregs and Teffs with similar TCRs, suggesting that self-reactive Tregs and Teffs could traverse thymic selection with similar TCR affinities.^10^ Interestingly, the difference in TCR repertoire in the ubiquitous antigen model was more pronounced at the higher end of the TCR affinity spectrum, implying that negative selection of Teffs drove the difference in repertoires, rather than preferential positive selection of high-affinity Tregs.^10^ Consistently, ubiquitously expressed Cre antigen was shown to drive deletion of conventional T cells instead of Treg generation.^44^ Analogous observations were obtained in a fixed-TCR system, in which ectopic overexpression of insulin increased negative selection of high-affinity but not low-affinity T cells, while both TCRs supported Treg development.^45^ Our observations showing increased expression of CD5 and Nur77-GFP on Tregs in wild-type NOD mice and in TCR-limited P2-TCRα mice were consistent with the two-step instructive model (Figs. 1 and 2D, E). We also observed a decrease in Treg development in mice with mutated target insulin epitope, indicating a critical role for specific self-antigen in Treg development (Fig. S4C).^6^ However, the surprising finding in our study was the broad range of Treg TCR affinities and the presence of low-affinity Tregs in the autoimmune response. Nevertheless, we cannot exclude the possibility that the relatively high degree of TCR overlap and TCR similarities observed in our fixed TCR alpha model could be artificially augmented due to restricted repertoire.

While mutation of insulin epitope curbed Treg development (Fig. S3C), expanding Treg developmental niche using BM chimeras did not increase Treg frequencies (Fig. S4E, F). Our inability to increase Treg development by constraining the niche may reflect a relatively unsaturated status of the insulin-specific T cell niche. This is potentially due to systemic circulation of insulin, and capture, processing, and presentation of insulin by antigen-presenting cells in distal lymph nodes.^46^ Peripheral circulating dendritic cells contribute to thymic antigen presentation and could increase the niche for insulin Treg development.^46–49^ These observations might indicate that insulin antigen due to its systemic distribution provides a relatively large niche for thymic Treg development, allowing the development of low-affinity Tregs. Whether low-affinity Tregs specific for other beta cell antigens develop and are recruited into the pancreatic islets still needs to be resolved.

Our observations regarding higher level of TCR signaling in Tregs expressing the same TCR as effector T cells is intriguing and suggests a cell-intrinsic capacity of Tregs to augment responses to TCR/pMHC interaction (Fig. 5). The cell-intrinsic mechanisms that support higher Treg sensitivity for pMHC ligands is unclear. TCR affinity does not always predict T cell functional outcome.^50^ It is possible that for some antigens difference in Treg/Teff TCR repertoires is connected to structural differences in how Treg TCRs engage pMHC ligands.^51^ Biophysical parameters and coreceptors can also influence signal transmission in T cells and T cell recruitment into immune response.^52^ Therefore, multiple mechanisms could be implicated in driving the increased TCR signaling in Tregs, including TCR clustering, coreceptor tuning, or differences in the metabolic state of the cells. How this Treg-intrinsic quality is translated into improved Treg function in vivo is unknown, but it is tempting to postulate that it supports an increased ability of Tregs to compete for limiting amounts of pMHC ligands. However, while low-reactivity TR-13 Tregs were able to infiltrate pancreatic islets (Fig. 3E, H) and to compete for ligand as indicated by higher Nur77-GFP compared with TR-13 Teffs (Fig. 5E), TR-13 Tregs failed to suppress pathogenic 4-8 TCR effectors (Fig. 4B), suggesting that there are certain limitations to insulin-specific Treg TCR functional repertoire. Two possibilities should be considered for why low-reactivity Tregs are insufficient. We can postulate that low-affinity Tregs are not able to compete with high-affinity Teffs; alternatively, there is an intrinsic insufficiency in TCR signaling that prevents low-affinity Treg function. On one hand TR-13 Tregs can prevent TR-13 T cell recruitment into the pancreas (Fig. 5D), suggesting some level of function is present in low-reactivity TR-13. At the same time TR-13 Tregs do not persist or accumulate in the pancreas in a transfer system (Fig. 4F), suggesting potential functional deficiency in a competitive system with pathogenic 4-8 T cells. We have previously published that low-affinity Tregs can contribute to protection in T1D against a combination of low/high-affinity effectors.^17^ In that study, we used TCRs isolated from effector T cells, and it was not clear whether such low-affinity Tregs are naturally recruited to the pancreas. The current study has identified that low-affinity TCRs are naturally present within the Treg compartment. We have also published that islet-infiltrating polyclonal low-affinity Tregs, as defined by lower CD5 expression, exhibit reduced function compared with CD5 high Tregs.^16^ Rather than simply correlating with imcreased affinity, optimal Treg functionality may require a sweet spot of TCR activation. An important take-home message is that standard approaches used to compare TCR signaling between Teff and Treg populations, CD5 and Nur77-GFP, are not always reliable readouts of differences in TCR affinities.

Tregs were previously reported to have dampened TCR signaling, as measured by maximum Erk phosphorylation, compared with naïve T cells.^53^ However, our data suggest increased activation of downstream target genes Nur77 and Nor1 in Tregs (Fig. 5G–J), and increased in vivo induced Nur77-GFP expression in Tregs (Fig. 5E). These observations are consistent with a study that demonstrated a Treg specific pattern of TCR signaling component activation, including a higher level of ITAM phosphorylation at a steady state.^54^ Tonic level of TCR signaling in a ligand-independent manner has been observed in certain T cell populations, resulting in a basal level of expression of downstream immediate gene targets like *Nr4a1* (Nur77). The role of tonic or basal signaling in T cells has been linked to maintaining cellular metabolism, memory T cell survival, and keeping T cells primed for rapid response to agonist TCR stimulation.^55^^,^^56^ Increased Nur77 expression in Tregs in vivo is consistent with continuous dependence on TCR signaling for Treg function.^34^^,^^35^ Whether higher Nur77 in Tregs is driven in part by nonligand basal TCR signaling has not been resolved. When Tregs were cultured in the absence of TCR stimulation for 20 h, they lost Nur77 expression (Fig. 5I), suggesting that continuous TCR stimulation in response to agonist or low-affinity cross-reactive ligands is necessary to maintain Nur77 expression in Tregs. However, Tregs were also able to respond with higher Nur77 to anti-CD3 stimulation compared with Teffs when starting at the same low level of Nur77 expression. Further investigation is needed to resolve the relationship between regulation of TCR signaling, Treg sensitivity to antigen, and the ultimate impact on Treg suppressive function.

It is possible that for some antigens difference in Treg/Teff TCR repertoires might underlie structural differences of how Treg TCRs engage pMHC ligands, rather than TCR affinity. Structural analysis of a human Treg–derived TCR specific for an insulin peptide showed reversed polarity of TCR docking on pMHC compared with conventional T cell TCR.^51^ Ins-tet+ Treg TCRs seemed to prefer certain V-beta chains, suggesting Treg structural preferences. How TCR structures influence biophysical parameters of TCR/pMHC interactions in the context of a T cell membrane, TCR signaling complex, and engagement of the downstream pathways is still largely unresolved. Recently developed technologies, including bio-membrane force probe, allow some of these questions to be addressed in the context of a live cell membrane.^36^^,^^37^^,^^57^ Importantly, these approaches have exposed a significant contribution of specific V-beta CDR2 sequences in forming stable TCR/pMHC complex interaction.^58^ These observations increase the importance of V-beta chain usage which may predispose certain biophysical characteristics favored by Tregs.

In conclusion, our study provides a detailed analysis of TCR repertoires and TCR characteristics exhibited by Tregs recruited into the spontaneous autoimmune response in the context of a susceptible MHC allele. While some observations were consistent with previous studies, such as higher expression of CD5 and Nur77-GFP in Tregs, this increase in TCR signaling was not reflected in Treg TCR reactivity for insulin. Moreover, insulin-specific Treg TCRs failed to support better Treg development or function in inhibiting diabetes compared with Teff TCRs. This surprising diminished suppressive potency of Treg TCRs may contribute to the pathogenesis of diabetes in NOD mice. Overall, our study puts additional emphasis on the relative capacity of Treg and Teff cells to respond to self-antigen as the deciding factor in tolerance vs autoimmunity.

## Supplementary Material

vkag136_Supplementary_Data

## Data Availability

The datasets generated during and/or analyzed during the current study are available from the corresponding author on reasonable request.
