# Supplementary material for: Increased Nur77 is disconnected from TCR affinity in insulin-specific Tregs
Source: J Immunol. 2026 Jul 2;215(6):vkag136. doi: 10.1093/jimmun/vkag136 (PMC13326740; doi:10.1093/jimmun/vkag136)
Supplement: vkag136_Supplementary_Data [file vkag136_supplementary_data.zip › Supplemental Figures.pdf]

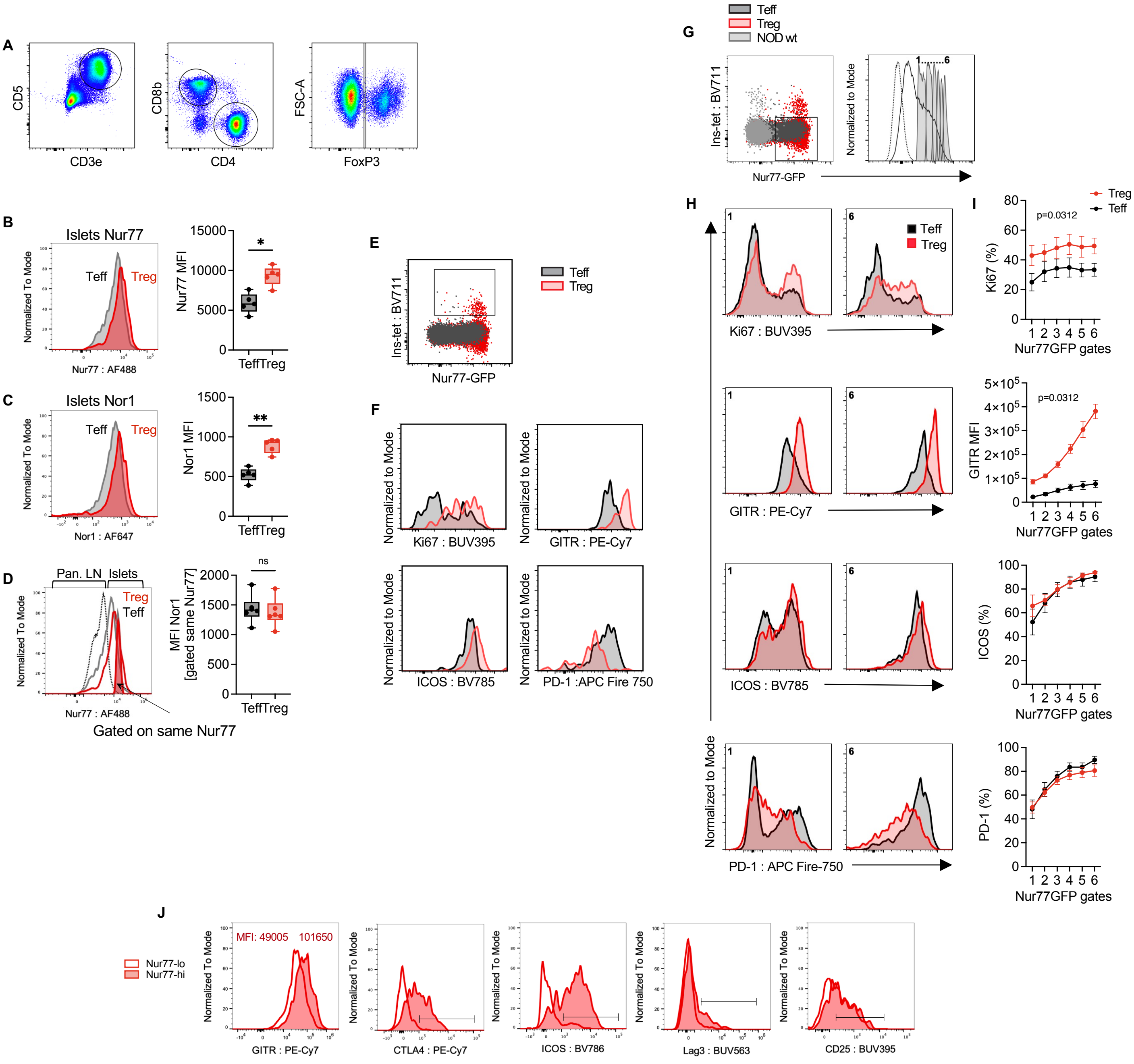

Supplementary Figure 1. Islet-infiltrating CD4+ Tregs show higher level of TCR signaling and activation compared to CD4+ Teffs. (**A-D**) CD4+ T cells from the islets of pre-diabetic 12-week-old NOD female mice (gating strategy in A) were stained for intracellular Nur77 (B) and Nor1 (C) and analyzed by flow cytometry. Analysis is gated on CD4+CD3+CD5+ (as shown in A) and then Foxp3+/-. Box and whiskers plots - each dot represents islet T cells from a single mouse. (**D**) Nor1 MFI in Teffs and Foxp3+ Tregs after gating on the same level of Nur77 expression. Solid grey (Teff) and red (Treg) overlapping histograms of narrow pre-gated Nur77 are backgated onto Nur77 expression in pancreatic lymph node CD4+CD3+CD5+ cells (dash line), CD4+CD3+CD5+ Foxp3- effectors (solid grey line) and CD4+CD3+CD5+Foxp3+ Tregs (solid red line) in the islets of NOD mice. (**E-I**) Ins-tet+ and Ins-tet- CD4+ Teffs and Tregs from the islets of 12-15 NOD.Nur77-GFP pre-diabetic female mice, n=6. (E) Gating strategy for Ins-tet+ cells. (F) Representative flow plots for Ins-tet+ Tregs and Teffs. (G) Gating strategy for Nur77-GFP+ Ins-tet- cells. (H) Representative histograms for lowest (1) and highest (6) gates of Nur77-GFP expression. (I) Ki67, GITR, ICOS and PD-1 expression on Tregs (red) and Teffs (black) subgated on levels of Nur77 as in G. (**J**) Representative histograms for Figure 1I showing Tregs analysis in the islets of pre-diabetic 12-week-old NOD female mice. Statistical analysis was performed using Mann-Whitney, *p<0.05; **p<0.01, ns>0.05.

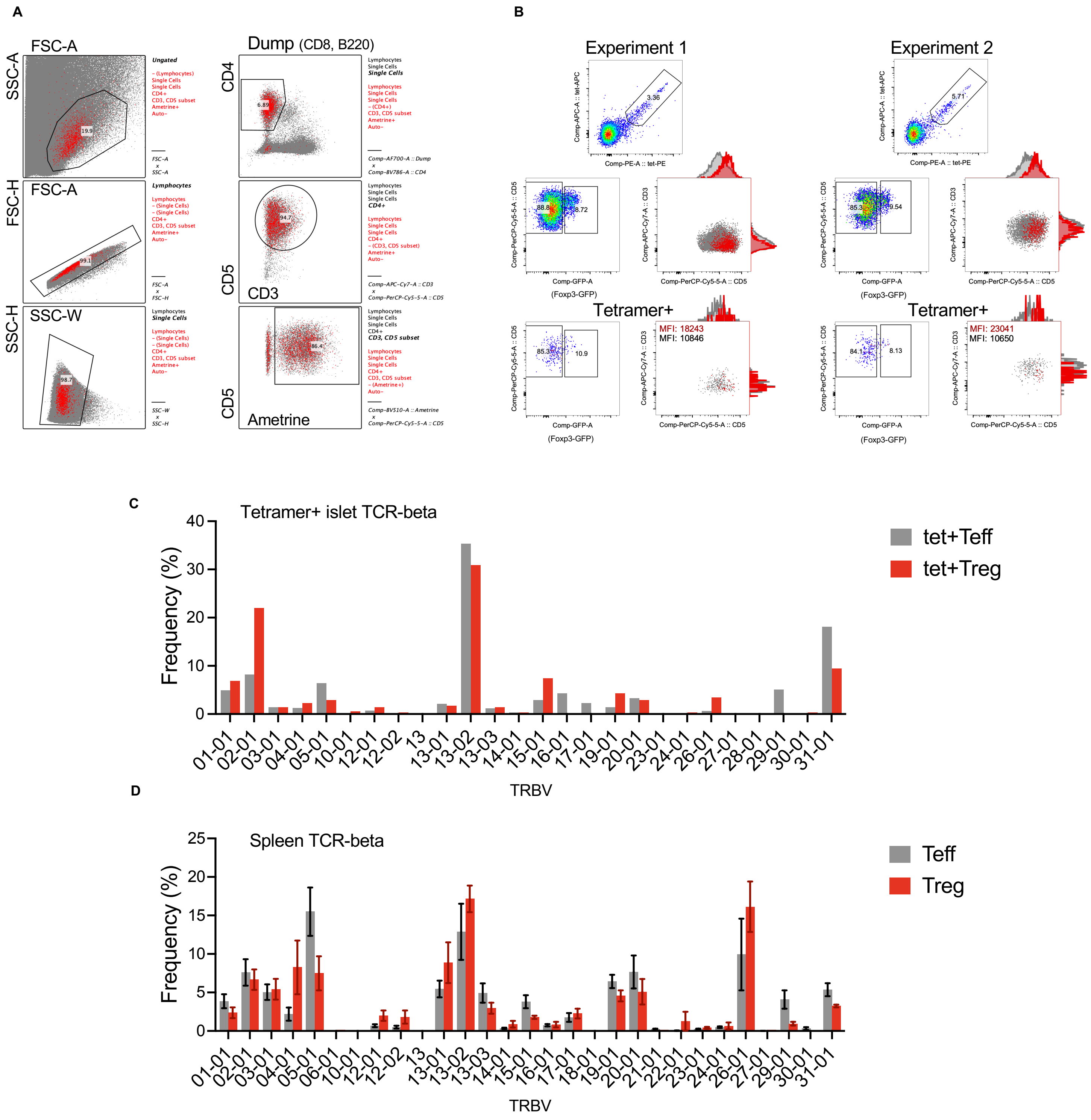

Supplementary Figure 2. TRBV gene usage in spleen and Ins-tet+ islet repertoires of P2-TCRα mice. (**A-B**) Cell sorting gating strategy for TCR-P2a tetramer+ Tregs (GFP+) and Teffs (GFP-). (**B**) Tetramer and CD5 expression are shown from two separate experiments. (**C-D**) 38 P2-TCRα mice were sacrificed 10-12 wk after BM transfer. Ins-tet+ cells from pancreatic islets were sorted, pooled, and sequenced. Tregs and Teffs were also sorted from 5 individual spleens without tetramer staining and were sequenced separately. TRBV gene usage of (**C**) islet Ins-tet+ repertoires and (**D**) splenic repertoires are shown. In (**D**), data are plotted as means ± SEM (n=5) and analyzed by paired multiple t test.

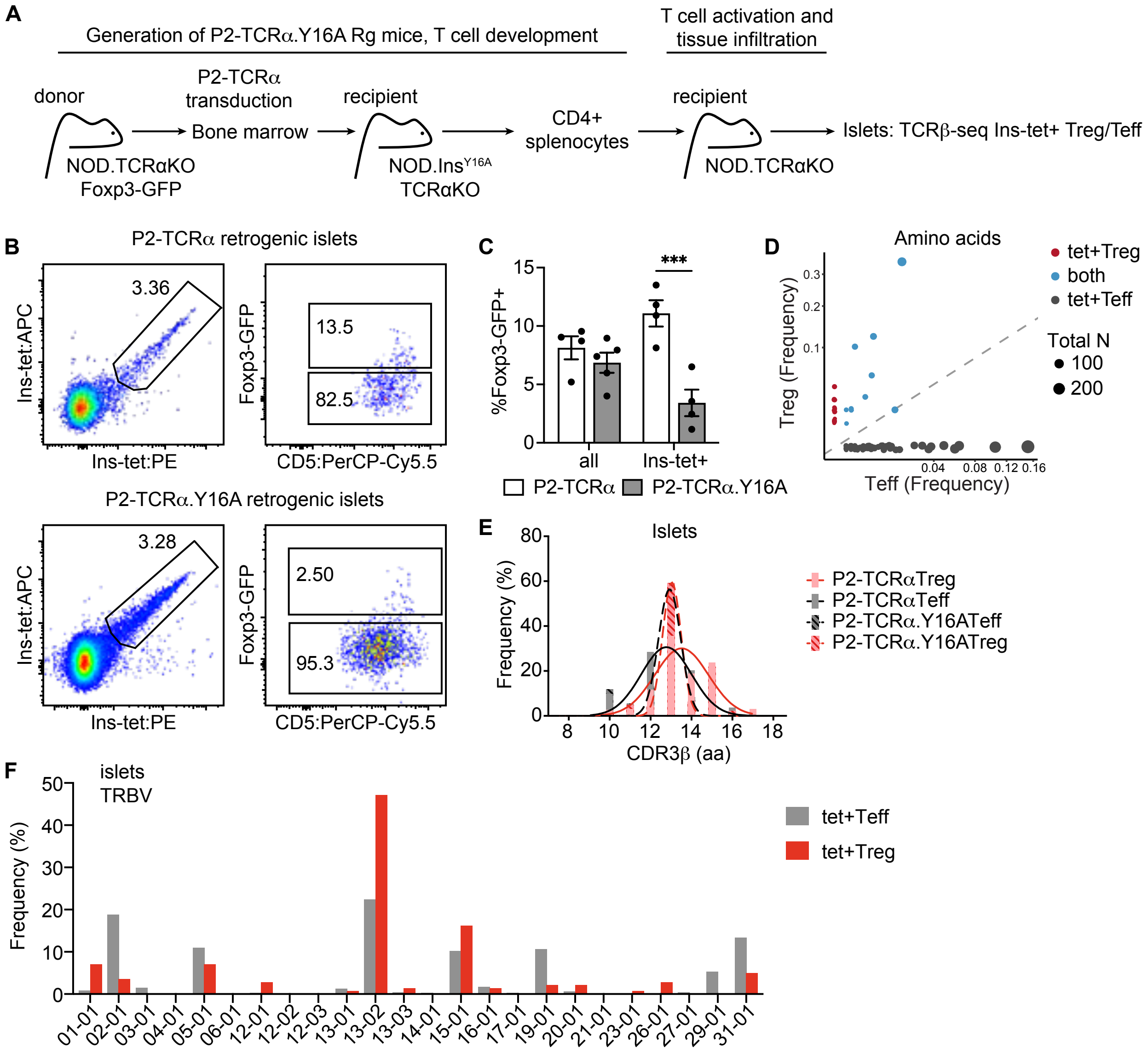

Supplementary Figure 3. TCRβ repertoire analysis of islet Ins-tet+ Tregs and Teffs of P2-TCRα.Y16A mice. (A) Schematic diagram for generation of P2-TCRα.Y16A retrogenic mice and Ins-tet+ TCRβ repertoire analysis. BM cells of NOD.TCRαKO.Foxp3-GFP mice were transduced with a retroviral vector that encoded the TCRα chain of TCR P2 and Ametrine fluorescent reporter. Transduced BM cells were transferred (IV) into NOD. Ins^{Y16A}.TCRαKO mice. Splenic CD4+ T cells were isolated from BM recipients 8-10wk after BM transfer and transferred (IP) into NOD.TCRαKO mice. 10-12 weeks later, pancreatic islet-infiltrating Treg and Teff cells were isolated and stained with Ins-tet. TCRβ repertoires of Ins-tet+ Tregs and Teffs were sequenced and analyzed. (B, C) Ins-tet staining and frequencies of Foxp3-GFP+ Tregs within Ins-tet+ populations or all islet CD4+ T cells (all). Data are plotted as means ± SEM (n=4 or 5) and analyzed by two-way ANOVA and Benjamini, Krieger and Yekutieli test. (D) Repertoire overlap between islet infiltrating Ins-tet+ Tregs and Teffs at amino acid level. Dot size represents the size of clonotypes. (E) Distributions of CDR3β length. Extra sum-of-squares F test. (F) TRBV gene usage. ***, p ≤ 0.001; ns > 0.05.

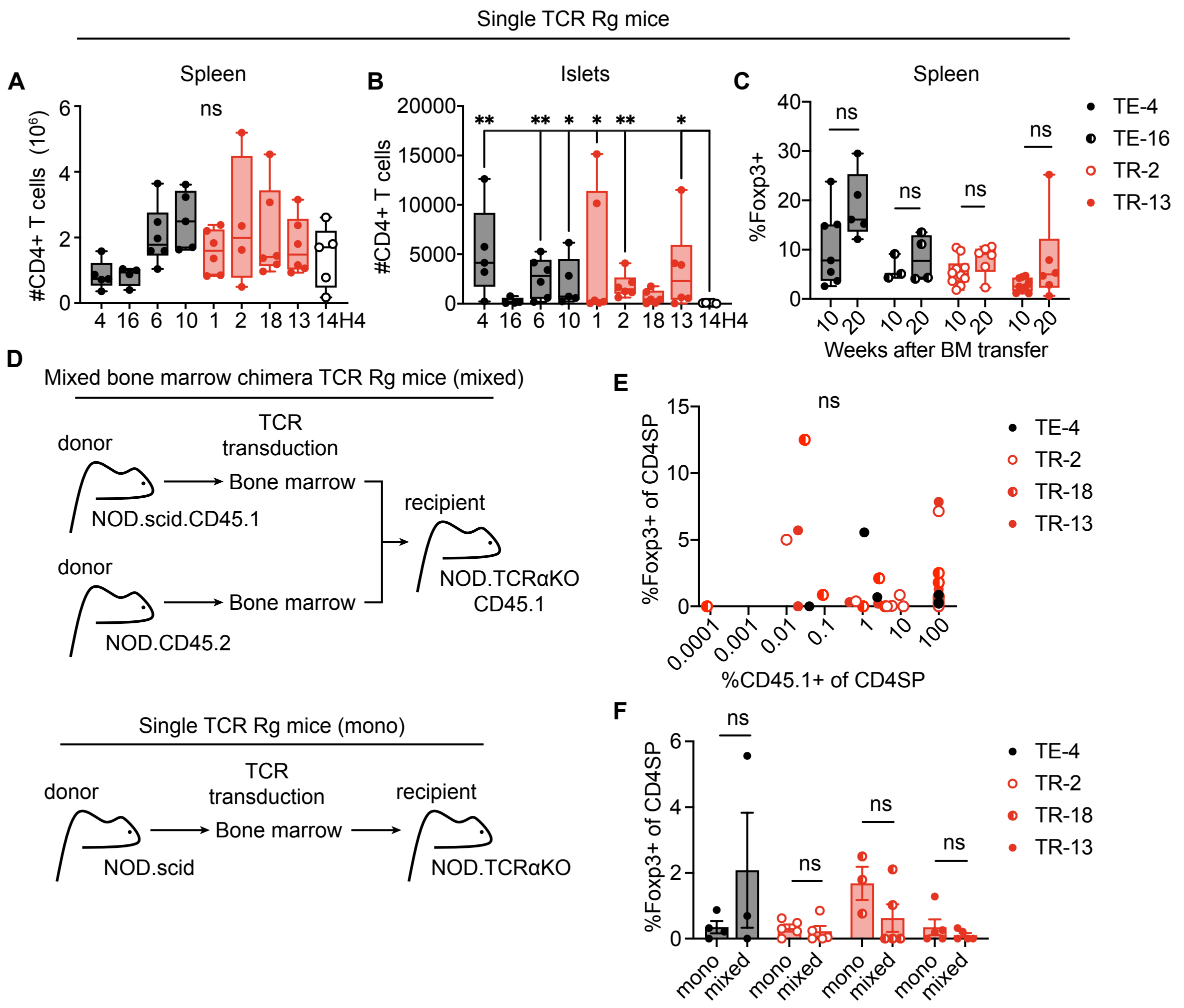

Supplementary Figure 4. Supplementary data to Figure 4. (A-C) Retrogenic mice expressing Teff (black), Treg (red) derived TCRs, or HEL specific 14H4 control TCR (white) were generated by transferring transduced NOD.scid BM (IV) into irradiated NOD.TCRαKO recipients. (A) Numbers of CD4+ T cells in spleens. (B) Numbers of Tregs in pancreatic islets. Mice were analyzed 20wk after BM transfer or at diabetes onset. (C) Frequencies of Tregs in spleens of TCR Rg mice analyzed 10wk or 20wk after BM transfer. (D-F) TCR transduced NOD.scid BM were transferred alone (mono) or co-transferred with NOD.CD45.2 BM (mixed) intravenously into NOD.TCRαKO mice. Recipients were sacrificed 5-7wk after BM transfer. (E) Thymic TCR retrogenic Treg frequencies are plotted against frequencies of CD45.1+ cells in CD4SP thymocytes. (F) Thymic Foxp3+ frequencies within TCR transduced immature CD4SP thymocytes (CD45.1+CD4+CD8-CD3+CD5+CD73-). Data are plotted as means ± SEM (n=3-10). ns > 0.05 by (A and B) one-way ANOVA followed by Benjamini, Krieger and Yekutieli's test (all comparisons are made with 14H4 negative control), (C) multiple Welch t test with Benjamini, Krieger and Yekutieli's correction. (E) Pearson correlation and (F) Welch t test. Outliers were identified and removed from analysis using ROUT method (Q=1).

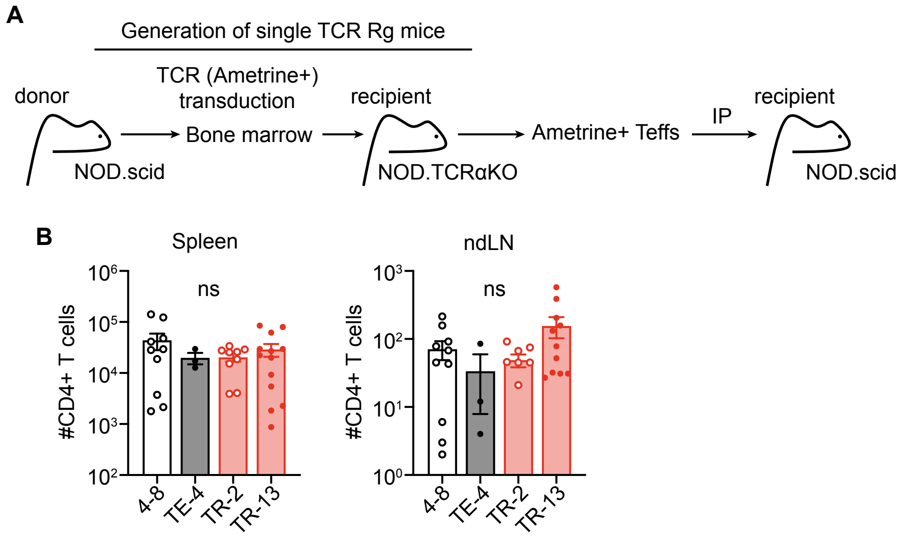

Supplementary Figure 5. Retrogenic Teffs expressing TCR with low insulin reactivity did not show reduced proliferation upon transfer. (A) Teffs from spleens and ndLNs of TCR Rg mice were sorted and transferred (IP) into NOD.scid female recipients. Recipients were sacrificed for analysis 16wk after transfer or at diabetes onset. (B) Numbers of donor T cells in spleens and ndLNs. Data were pooled from two independent experiments. ns > 0.05 by Kruskal-Wallis test followed by Dunn's test (n=3-11).
