# Supplementary material for: Increased Nur77 is disconnected from TCR affinity in insulin-specific Tregs
Source: J Immunol. 2026 Jul 2;215(6):vkag136. doi: 10.1093/jimmun/vkag136 (PMC13326740; doi:10.1093/jimmun/vkag136)
Supplement: vkag136_Supplementary_Data [file vkag136_supplementary_data.zip › Supplementary Tables.docx]

|  | Teff | | Treg | | Shared | | %Shared of Teff | | %Shared of Treg | |
| --- | --- | --- | --- | --- | --- | --- | --- | --- | --- | --- |
|  | Amino acid | DNA | Amino acid | DNA | Amino acid | DNA | Amino acid | DNA | Amino acid | DNA |
| Ins-tet+ islets | | | | | | | | | | |
| P2-TCRα | 358 | 584 | 92 | 118 | 35 | 32 | 9.8% | 5.5% | 38.0% | 27.1% |
| P2-TCRα.Y16A | 210 | 267 | 35 | 41 | 10 | 11 | 4.8% | 4.1% | 28.6% | 26.8% |
| Spleens | | | | | | | | | | |
| P2-TCRα | 1189.6 | 1818.4 | 177.4 | 481.6 | 148 |  | 11.2±2.2 |  | 48.4±8.6 |  |

**Supplementary Table I. Summary of Treg and Teff TCRβ repertoire sequencing**

Numbers of unique TCRβ sequences, and repertoire overlap between islet-infiltrating Ins-tet+ Tregs and Teffs that develop in P2-TCRα fixed alpha-chain mice, or in insulin epitope mutant P2-TCRα.Y16A mice.

**Ins-tet+ islet P2-TCRα:** Samples were sorted and combined from 38 mice at 10-12wk post BM transfer.

**Ins-tet+ islet P2-TCRα.Y16A**: Islet-infiltrating Ins-tet+ Treg and Teff cells were sorted from 25 splenic CD4+ T cell recipients generated using 7 P2-TCRα.Y16A donors.

**Spleen P2-TCRα:** Samples were sorted from separate spleens of 5 recipient mice. Analysis shown is an average of 5 samples.

**Supplementary Table II. Selected Ins-tet+ positive Treg and Teff TCRs from pancreatic islets of P2-TCRα retrogenic mice.**

|  | | CDR3β (aa) | Vβ | Dβ | Jβ | Treg/Teff | Sp Teff | Sp Treg | 4G4  IL-2 | IEDB |
| --- | --- | --- | --- | --- | --- | --- | --- | --- | --- | --- |
| Teff TCRs | 4 | CASGGWGGNTLYF | 13-02*01 | 02-01*01 | 02-04*01 | 0.00 | 1 | 0 | yes |  |
|  | 6 | CASRDWGDEQYF | 15-01*01 | 02-01*01 | 02-07*01 | 0.00 | 2 | 0 | yes |  |
|  | 7 | CASSAKTNSDYTF | 13-03*01 | unknown | 01-02*01 | 0.00 | 1 | 0 |  | Ins-2 |
|  | 10 | CASSPGQGSEQYF | 05-01*01 | 01-01*01 | 02-07*01 | 0.00 | 1 | 0 | yes | Ins-1/2 |
|  | 14 | CASSSGGQGYEQYF | 17-01*01 | 01-01*01 | 02-07*01 | 0.00 | 1 | 0 | yes | Ins-2 |
|  | 15 | CASSSRRQPYEQYF | 16-01*01 | 01-01*01 | 02-07*01 | 0.00 | 1 | 0 |  |  |
|  | 16 | CAWKGDRLFF | 31-01*01 | 01-01*01 | 01-04*01 | 0.00 | 2 | 0 | yes |  |
|  | 17 | CAWSLTGGGIEQYF | 31-01*01 | 02-01*01 | 02-07*01 | 0.00 | 0 | 0 | yes |  |
|  | 19 | CTCSADTGGEQYF | 01-01*01 | 01-01*01 | 02-07*01 | 0.00 | 2 |  | yes | Ins-2 |
| Treg TCRs | 1 | CASADWGGNTLYF | 13-02*01 | 02-01*01 | 02-04*01 | 1.65 | 2 | 2 | yes |  |
|  | 2 | CASGDWGGNTLYF | 13-02*01 | 02-01*01 | 01-03*01 | 1.23 | 3 | 0 | yes |  |
|  | 3 | CASGEAWGGAEQYF | 13-02*01 | 02-01*01 | 02-07*01 | 0.56 | 5 | 5 |  |  |
|  | 5 | CASGLRDRGDTQYF | 13-02*01 | 01-01*01 | 02-05*01 | 1.50 | 5 | 5 |  |  |
|  | 8 | CASSLRPRGDSGNTLYF | 26-01*01 | 01-01*01 | 01-03*01 | - | 1 | 1 |  |  |
|  | 9 | CASSPGDSPLYF | 15-01*01 | 01-01*01 | 01-06*01 | 1.00 | 3 | 3 |  | Ins-2 |
|  | 11 | CASSQDGTEVFF | 02-01*01 | 02-01*01 | 01-01*01 | - | 4 | 3 |  | Ins-2 |
|  | 13 | CASSQGQGYEQYF | 02-01*01 | 01-01*01 | 02-07*01 | 0.14 | 0 | 0 | yes | Ins-1/2 |
|  | 18 | CGASGYNNQAPLF | 20-01*01 | unknown | 01-05*01 | - | 1 | 0 | yes | Ins-2 |

**Treg/Teff:** ratios of templates of Treg verses Teff in islets.

**Sp Teff, Sp Treg:** Number of mice that harbored the TCR clonotype in splenic Teff or Treg compartment, respectively.

**4G4 IL-2:** Reactivity to insulin peptide shown in Figure 2K and 2L.

**IEDB:** Antigen specificity predicted by IEDB TCRMatch, details in Supplementary Tables III and IV.

**Supplementary Table III. CDR3β amino acid sequences with predicted insulin reactivity.**

| Sample | Population | Unique clone | TCRMatch | %Matched |
| --- | --- | --- | --- | --- |
| P2-TCRa | Teff | 358 | 103 | 29% |
|  | Treg | 92 | 32 | 35% |

Islet-derived P2-TCRα retrogenic CDR3-beta sequences were queried using the IEDB TCRMatch Tool with a threshold of 0.90 for antigen specificity prediction based on sequence similarity. See Supplementary Table V for details.

**Supplementary Table IV. CDR3β amino acid sequences identified from the pancreatic islets of P2-TCRα retrogenic mice with direct match to public databases.**

| Sample | Population | CDR3b | Productive frequency | Antigen | Database | Reference ID |
| --- | --- | --- | --- | --- | --- | --- |
| P2-TCRα | Teff | CASSQDSNSPLYF | 0.0010 | Insulin-2 | MacPAS-TCR; IEDB | 31471352; 35146 |
| P2-TCRα | Teff | CASSDSAETLYF | 0.0022 | Insulin-2 | MacPAS-TCR; IEDB | 31471352; 35032 |
| P2-TCRα | Teff | CASSRLGSAETLYF | 0.0022 | Insulin-2 | IEDB | 35172 |

CDR3β sequences of insulin tetramer binding Treg and Teff cells isolated from pancreatic islets of P2-TCRα retrogenic mice were searched against public TCR databases, including MacPAS-TCR and IEDB. Three CDR3β sequences were identified as direct matches to insulin reactive TCRs available in the databases. Reference ID, PMID or IEDB receptor group.

**Supplementary Table V. CDR3b amino acid sequences identified from the pancreatic islets of P2-TCRa retrogenic mice with predicted insulin reactivity.**

| Name | Query | Matched sequence | Match score | Receptor group | Antigen | Database |
| --- | --- | --- | --- | --- | --- | --- |
| TR9 | ASSPGDSPLY | ASSPRESPLY | 0.91 | 35063 | Insulin-2 | IEDB |
| TR11 | ASSQDGTEVF | ASSQERTEVF | 0.92 | 35068 | Insulin-2 | IEDB |
|  | ASSQDGTEVF | ASSFSQGTEVF | 0.90 | 35034 | Insulin-2 | IEDB |
| TR13 | ASSQGQGYEQY | ASSAGQGYEQY | 0.96 | 75802 | non-structural protein NS4b,Insulin-1,ORF3a protein [Severe acute respiratory syndrome coronavirus 2] | IEDB |
|  | ASSQGQGYEQY | ASSQGQYEQY | 0.92 | 56812 | Insulin-1 | IEDB |
|  | ASSQGQGYEQY | ASSVGGQGYEQY | 0.91 | 35176 | Insulin-2 | IEDB |
|  | ASSQGQGYEQY | ASSQDQAYEQY | 0.91 | 56774 | Insulin-1 | IEDB |
|  | ASSQGQGYEQY | ASSLQGGYEQY | 0.90 | 35056 | Insulin-2 | IEDB |
|  | ASSQGQGYEQY | ASSQGGEQY | 0.90 | 58141 | Insulin-1 | IEDB |
| TR18 | GASGYNNQAPL | ASSGNNQAPL | 0.91 | 35116 | Insulin-2 | IEDB |
| TE7 | ASSAKTNSDYT | ASSQDTNSDYT | 0.93 | 35149 | Insulin-2 | IEDB |
|  | ASSAKTNSDYT | ASSRDTNSDYT | 0.93 | 35048 | Insulin-2 | IEDB |
|  | ASSAKTNSDYT | ASGRDTNSDYT | 0.91 | 35028 | Insulin-2 | IEDB |
|  | ASSAKTNSDYT | ASGDVTNSDYT | 0.91 | 35101 | Insulin-2 | IEDB |
|  | ASSAKTNSDYT | ASSQDINSDYT | 0.91 | 35141 | Insulin-2 | IEDB |
| TE10 | ASSPGQGSEQY | ASSAGQGYEQY | 0.92 | 75802 | non-structural protein NS4b,Insulin-1,ORF3a protein [Severe acute respiratory syndrome coronavirus 2] | IEDB |
|  | ASSPGQGSEQY | ASSPGQGRAPL | 0.90 | 35134 | Insulin-2 | IEDB |
|  | ASSPGQGSEQY | ASSPGQGNTEVF | 0.90 | 35061 | Insulin-2 | IEDB |
|  | ASSPGQGSEQY | ASSQGGEQY | 0.90 | 58141 | Insulin-1 | IEDB |
| TE14 | ASSSGGQGYEQY | ASSVGGQGYEQY | 0.96 | 35176 | Insulin-2 | IEDB |
|  | ASSSGGQGYEQY | ASSAGQGYEQY | 0.95 | 75802 | non-structural protein NS4b,Insulin-1,ORF3a protein [Severe acute respiratory syndrome coronavirus 2] | IEDB |
|  | ASSSGGQGYEQY | ASSVGGQGYEQY | 0.96 | 35176 | Insulin-2 | IEDB |
| TE19 | TCSADTGGEQY | TCSAGTGIEQY | 0.91 | 35086 | Insulin-2 | IEDB |
| - | ASSSGQGYEQY | ASSAGQGYEQY | 0.98 | 75802 | non-structural protein NS4b,Insulin-1,ORF3a protein [Severe acute respiratory syndrome coronavirus 2] | IEDB |
| - | ASSQDTNSPLY | ASSQDSNSPLY | 0.98 | 35146 | Insulin-2 | IEDB |
| - | ASSRLGSAETLY | ASGRLGSAETLY | 0.97 | 35043 | Insulin-2 | IEDB |
| - | ASSRLGSAETLY | ASDRLGSAETLY | 0.97 | 35025 | Insulin-2 | IEDB |
| - | ASSPDNSGNTLY | ASSPDSSGNTLY | 0.97 | 56738 | Insulin-1 | IEDB |
| - | ASSQDENSPLY | ASSQDSNSPLY | 0.97 | 35146 | Insulin-2 | IEDB |
| - | ASSTSAETLY | ASSDSAETLY | 0.96 | 35032 | Insulin-2 | IEDB |
| - | ASSIGQGYEQY | ASSAGQGYEQY | 0.96 | 75802 | non-structural protein NS4b,Insulin-1,ORF3a protein [Severe acute respiratory syndrome coronavirus 2] | IEDB |
| - | ASSPDGSGNTLY | ASSPDSSGNTLY | 0.96 | 56738 | Insulin-1 | IEDB |
| - | ASSQDVNSPLY | ASSQDSNSPLY | 0.96 | 35146 | Insulin-2 | IEDB |
| - | ASSAGTGNTQY | ASSAGSGNTLY | 0.95 | 35031 | Insulin-2 | IEDB |
| - | ASSQDMNSPLY | ASSQDSNSPLY | 0.95 | 35146 | Insulin-2 | IEDB |
| - | ASSPGQGYEQY | ASSAGQGYEQY | 0.95 | 75802 | non-structural protein NS4b,Insulin-1,ORF3a protein [Severe acute respiratory syndrome coronavirus 2] | IEDB |
| - | ASSQDLNSPLY | ASSQDSNSPLY | 0.95 | 35146 | Insulin-2 | IEDB |
| - | ASSQDPNSPLY | ASSQDSNSPLY | 0.95 | 35146 | Insulin-2 | IEDB |
| - | ASSQDPNSPLY | ASSQDSNSPLY | 0.95 | 35146 | Insulin-2 | IEDB |
| - | ASGDWGGLYEQY | ASGDWGGAYEQY | 0.95 | 56667 | Insulin-1 | IEDB |
| - | GARAGAGNTLY | ASTAGAGNTLY | 0.95 | 35179 | Insulin-2 | IEDB |
| - | ASSGGVGNTLY | ASSGGVQNTLY | 0.95 | 35115 | Insulin-2 | IEDB |
| - | ASSLAGQGYEQY | ASSVGGQGYEQY | 0.95 | 35176 | Insulin-2 | IEDB |
| - | ASSQDHNSPLY | ASSQDSNSPLY | 0.95 | 35146 | Insulin-2 | IEDB |
| - | ASSRAGQGYEQY | ASSAGQGYEQY | 0.95 | 75802 | non-structural protein NS4b,Insulin-1,ORF3a protein [Severe acute respiratory syndrome coronavirus 2] | IEDB |
| - | ASSLAGQGYEQY | ASSAGQGYEQY | 0.94 | 75802 | non-structural protein NS4b,Insulin-1,ORF3a protein [Severe acute respiratory syndrome coronavirus 2] | IEDB |
| - | ASSDSGDSDYT | ASSFSGDSDYT | 0.94 | 35044 | Insulin-2 | IEDB |
| - | ASSQGGGQDTQY | ASSQGQGQDTQY | 0.94 | 35163 | Insulin-2 | IEDB |
| - | ASSQGGGQDTQY | ASSQGQGQDTQY | 0.94 | 35163 | Insulin-2 | IEDB |
| - | ASSGGVGNTLY | ASSAGSGNTLY | 0.94 | 35031 | Insulin-2 | IEDB |
| - | ASSLENERLF | ASQISNERLF | 0.94 | 35029 | Insulin-2 | IEDB |
| - | TCSARQGNTGQLY | ASSRQGNTGQLY | 0.94 | 35174 | Insulin-2 | IEDB |
| - | GARTGAGNTLY | ASTAGAGNTLY | 0.94 | 35179 | Insulin-2 | IEDB |
| - | ARGNNQAPL | ASSGNNQAPL | 0.94 | 35116 | Insulin-2 | IEDB |
| - | ASSAGTGNTQY | ASTAGAGNTLY | 0.94 | 35179 | Insulin-2 | IEDB |
| - | ASSQDGQGNTLY | ASSQQGSGNTLY | 0.94 | 35072 | Insulin-2 | IEDB |
| - | ASSGGVGNTLY | ASTAGAGNTLY | 0.93 | 35179 | Insulin-2 | IEDB |
| - | ASSLVGQGYEQY | ASSVGGQGYEQY | 0.93 | 35176 | Insulin-2 | IEDB |
| - | ASSQDGQGNTLY | ASSQEGQGETLY | 0.93 | 56792 | Insulin-1 | IEDB |
| - | ASSDQSSAETLY | ASSPRSSAETLY | 0.93 | 35136 | Insulin-2 | IEDB |
| - | ASRRQGSAETLY | ASSRLGSAETLY | 0.93 | 35172 | Insulin-2 | IEDB |
| - | ASRQGQGYEQY | ASSAGQGYEQY | 0.93 | 75802 | non-structural protein NS4b,Insulin-1,ORF3a protein [Severe acute respiratory syndrome coronavirus 2] | IEDB |
| - | ASSDQSSAETLY | ASSDSAETLY | 0.93 | 35032 | Insulin-2 | IEDB |
| - | GARAGAGNTLY | ASSAGSGNTLY | 0.93 | 35031 | Insulin-2 | IEDB |
| - | ASSRAGQGYEQY | ASSVGGQGYEQY | 0.93 | 35176 | Insulin-2 | IEDB |
| - | ASSLEGQYEQY | ASSLQGGYEQY | 0.93 | 35056 | Insulin-2 | IEDB |
| - | ASSQDAGGGYEQY | ASSQDRGGGFEQY | 0.93 | 56776 | Insulin-1 | IEDB |
| - | ASSDGGGEYEQY | ASSDAGGRYEQY | 0.93 | 35112 | Insulin-2 | IEDB |
| - | ASSQDTNSPLY | ASSQDTNSDYT | 0.93 | 35149 | Insulin-2 | IEDB |
| - | ASSLEGQYEQY | ASSQGQYEQY | 0.93 | 56812 | Insulin-1 | IEDB |
| - | ASSSGTGVEQF | TCSAGTGIEQY | 0.93 | 35086 | Insulin-2 | IEDB |
| - | TCSAGQSYEQY | ASSAGQGYEQY | 0.93 | 75802 | non-structural protein NS4b,Insulin-1,ORF3a protein [Severe acute respiratory syndrome coronavirus 2] | IEDB |
| - | ASSPGQGNERLF | ASSPGQGNTEVF | 0.93 | 35061 | Insulin-2 | IEDB |
| - | ASSSGQGYEQY | ASSVGGQGYEQY | 0.93 | 35176 | Insulin-2 | IEDB |
| - | ASSQDPAGNTLY | ASSQDTSGNTLY | 0.92 | 35150 | Insulin-2 | IEDB |
| - | ASSRGTGYEQY | ASSAGQGYEQY | 0.92 | 75802 | non-structural protein NS4b,Insulin-1,ORF3a protein [Severe acute respiratory syndrome coronavirus 2] | IEDB |
| - | ASSPGGGNTLY | ASSAGSGNTLY | 0.92 | 35031 | Insulin-2 | IEDB |
| - | ASSGGGSNERLF | ASGEGGTNERLF | 0.92 | 35102 | Insulin-2 | IEDB |
| - | ASSIGQGYEQY | ASSVGGQGYEQY | 0.92 | 35176 | Insulin-2 | IEDB |
| - | ASGLQGYEQY | ASILSGYEQY | 0.92 | 56671 | Insulin-1 | IEDB |
| - | ASRTGGANTLY | ASTAGAGNTLY | 0.92 | 35179 | Insulin-2 | IEDB |
| - | ASSDAGGASNERLF | ASSQEGGAANERLF | 0.92 | 56787 | Insulin-1 | IEDB |
| - | ASSLGEGTEVF | ASSFSQGTEVF | 0.92 | 35034 | Insulin-2 | IEDB |
| - | ASSLENERLF | ASSQISNERLF | 0.92 | 187558 | Insulin-2 | IEDB |
| - | ASSRGQGVEQY | ASSAGQGYEQY | 0.92 | 75802 | non-structural protein NS4b,Insulin-1,ORF3a protein [Severe acute respiratory syndrome coronavirus 2] | IEDB |
| - | ASSLRGGEQY | ASSQGGEQY | 0.92 | 58141 | Insulin-1,non-structural protein NS4b | IEDB |
| - | ASRRQGSAETLY | ASDRLGSAETLY | 0.92 | 35025 | Insulin-2 | IEDB |
| - | ASSPDNSGNTLY | ASSQDTSGNTLY | 0.92 | 35150 | Insulin-2 | IEDB |
| - | ASSLGQGIEQY | ASSAGQGYEQY | 0.92 | 75802 | non-structural protein NS4b,Insulin-1,ORF3a protein [Severe acute respiratory syndrome coronavirus 2] | IEDB |
| - | ASSGTVSNERLF | ASSQISNERLF | 0.92 | 187558 | Insulin-2 | IEDB |
| - | ASSQDGQGNTLY | ASSQDTSGNTLY | 0.92 | 35150 | Insulin-2 | IEDB |
| - | ASSQGQGTEVF | ASSFSQGTEVF | 0.92 | 35034 | Insulin-2 | IEDB |
| - | ASSGTGDAEQF | ASSAGTGDYEQY | 0.92 | 35108 | Insulin-2 | IEDB |
| - | ASRLGSQDTQY | ASSLGSQNTLY | 0.92 | 187279 | pM45,Insulin-2 | IEDB |
| - | ASRLGSQDTQY | ASSLGSQNTLY | 0.92 | 187279 | pM45,Insulin-2 | IEDB |
| - | ASSGTVSNERLF | ASTSTGISNERLF | 0.92 | 35077 | Insulin-2 | IEDB |
| - | GARTGAGNTLY | ASSAGSGNTLY | 0.92 | 35031 | Insulin-2 | IEDB |
| - | ASSLVGQGYEQY | ASSAGQGYEQY | 0.92 | 75802 | non-structural protein NS4b,Insulin-1,ORF3a protein [Severe acute respiratory syndrome coronavirus 2] | IEDB |
| - | ASRLGSQDTQY | ASSLQGQDTQY | 0.92 | 56717 | Insulin-1 | IEDB |
| - | ASRLGSQDTQY | ASSLQGQDTQY | 0.92 | 56717 | Insulin-1 | IEDB |
| - | GARQGAGNTLY | ASTAGAGNTLY | 0.92 | 35179 | Insulin-2 | IEDB |
| - | GARQGAGNTLY | ASTAGAGNTLY | 0.92 | 35179 | Insulin-2 | IEDB |
| - | ASRRQGSAETLY | ASGRLGSAETLY | 0.92 | 35043 | Insulin-2 | IEDB |
| - | ASSPGQEQY | ASSQGGEQY | 0.92 | 58141 | Insulin-1,non-structural protein NS4b | IEDB |
| - | ASSPGQEQY | ASSQGGEQY | 0.92 | 58141 | Insulin-1,non-structural protein NS4b | IEDB |
| - | ASRSGASSYEQY | ASSSGTGASSYEQY | 0.92 | 35175 | Insulin-2 | IEDB |
| - | ASSQDVNSPLY | ASSQDINSDYT | 0.92 | 35141 | Insulin-2 | IEDB |
| - | ASSIGQGYEQY | ASSLQGGYEQY | 0.92 | 35056 | Insulin-2 | IEDB |
| - | ASSSGTGVEQF | ASSPTGVEQY | 0.92 | 187528 | Insulin-2 | IEDB |
| - | TCSPGLGNEQY | SPGLGNEQY | 0.92 | 27742 | Insulin,Insulin-2, Chromogranin-A, | IEDB |
| - | TCSPGLGNEQY | SPGLGNEQY | 0.92 | 27742 | Insulin,Insulin-2, Chromogranin-A, | IEDB |
| - | TCSPGLGNEQY | SPGLGNEQY | 0.92 | 27742 | Insulin,Insulin-2, Chromogranin-A, | IEDB |
| - | ASSPGQGLEQY | ASSAGQGYEQY | 0.92 | 75802 | non-structural protein NS4b,Insulin-1,ORF3a protein [Severe acute respiratory syndrome coronavirus 2] | IEDB |
| - | ASSPGQGLEQY | ASSAGQGYEQY | 0.92 | 75802 | non-structural protein NS4b,Insulin-1,ORF3a protein [Severe acute respiratory syndrome coronavirus 2] | IEDB |
| - | ASSQGQNTEVF | ASSQGQTNQA | 0.92 | 56811 | Insulin-1 | IEDB |
| - | ASSQGQNTEVF | ASSQGQTNQA | 0.92 | 56811 | Insulin-1 | IEDB |
| - | ASSSQHYEQY | ASSQVYEQY | 0.92 | 35168 | Insulin-2 | IEDB |
| - | ASSPTTNSDYT | ASSQDTNSDYT | 0.92 | 35149 | Insulin-2 | IEDB |
| - | ASSPGQGTEVF | ASSPGQGNTEVF | 0.92 | 35061 | Insulin-2 | IEDB |
| - | ASSPGQGTEVF | ASSPGQGNTEVF | 0.92 | 35061 | Insulin-2 | IEDB |
| - | ASSPGQGTEVF | ASSPGQGNTEVF | 0.92 | 35061 | Insulin-2 | IEDB |
| - | ASSLARGYEQY | ASSLQGGYEQY | 0.92 | 35056 | Insulin-2 | IEDB |
| - | ASSDGGGEYEQY | ASSDAGGLYEQY | 0.91 | 35111 | Insulin-2 | IEDB |
| - | ASSDGGGEYEQY | ASSDAGGLYEQY | 0.91 | 35110 | Insulin-2 | IEDB |
| - | ASSDGGGEYEQY | ASSDAGGLYEQY | 0.91 | 35042 | Insulin-2 | IEDB |
| - | ASSQERGGEQY | ASSQEAGGNQA | 0.91 | 56784 | Insulin-1 | IEDB |
| - | ASSQAGGGSDYT | ASSQDTGGSDYT | 0.91 | 56781 | Insulin-1 | IEDB |
| - | ASGVQGYEQY | ASSQVGYEQY | 0.91 | 56696 | Insulin-1 | IEDB |
| - | ASGVQGYEQY | ASSQVGYEQY | 0.91 | 56696 | Insulin-1 | IEDB |
| - | ASGVQGYEQY | ASSQVGYEQY | 0.91 | 56696 | Insulin-1 | IEDB |
| - | ASSQGENTEVF | ASSQETGENTEVF | 0.91 | 35069 | Insulin-2 | IEDB |
| - | ASSLRLSNERLF | ASSQISNERLF | 0.91 | 187558 | Insulin-2 | IEDB |
| - | TCSARQGNTGQLY | ASSQQGNTGQLY | 0.91 | 56691 | Insulin-1 | IEDB |
| - | ASSQDSNSPLY | ASSQDTNSDYT | 0.91 | 35149 | Insulin-2 | IEDB |
| - | ASSFGQGIEQY | ASSAGQGYEQY | 0.91 | 75802 | non-structural protein NS4b,Insulin-1,ORF3a protein [Severe acute respiratory syndrome coronavirus 2] | IEDB |
| - | ASSPTTNSDYT | ASSRDTNSDYT | 0.91 | 35048 | Insulin-2 | IEDB |
| - | ASSTSAETLY | ASSQVTTSAETLY | 0.91 | 56698 | Insulin-1 | IEDB |
| - | ASSPDGSGNTLY | ASSAGSGNTLY | 0.91 | 35031 | Insulin-2 | IEDB |
| - | ASSQQEGEQY | ASSQGGEQY | 0.91 | 58141 | Insulin-1,non-structural protein NS4b | IEDB |
| - | ASRLGSQDTQY | ASSRGDQDTQY | 0.91 | 35171 | Insulin-2 | IEDB |
| - | ASRLGSQDTQY | ASSRGDQDTQY | 0.91 | 35171 | Insulin-2 | IEDB |
| - | ASRTGGANTLY | ASSAGSGNTLY | 0.91 | 35031 | Insulin-2 | IEDB |
| - | ASSSGQNTEVF | ASSFSQGTEVF | 0.91 | 35034 | Insulin-2 | IEDB |
| - | ASSPGGGNTLY | ASTAGAGNTLY | 0.91 | 35179 | Insulin-2 | IEDB |
| - | ASGGQNYAEQF | ASGRDNYAEQF | 0.91 | 35104 | Insulin-2 | IEDB |
| - | ASSQPGQGYEQY | ASSAGQGYEQY | 0.91 | 75802 | non-structural protein NS4b,Insulin-1,ORF3a protein [Severe acute respiratory syndrome coronavirus 2] | IEDB |
| - | ASSTSAETLY | ASSPRSSAETLY | 0.91 | 35136 | Insulin-2 | IEDB |
| - | ASSQGLGGQDTQY | GAGGLGGQDTQY | 0.91 | 35079 | Insulin-2 | IEDB |
| - | ASGVQGYEQY | ASSAGQGYEQY | 0.91 | 75802 | non-structural protein NS4b,Insulin-1,ORF3a protein [Severe acute respiratory syndrome coronavirus 2] | IEDB |
| - | ASGVQGYEQY | ASSAGQGYEQY | 0.91 | 75802 | non-structural protein NS4b,Insulin-1,ORF3a protein [Severe acute respiratory syndrome coronavirus 2] | IEDB |
| - | ASGVQGYEQY | ASSAGQGYEQY | 0.91 | 75802 | non-structural protein NS4b,Insulin-1,ORF3a protein [Severe acute respiratory syndrome coronavirus 2] | IEDB |
| - | ASSIVGVEQY | ASSPTGVEQY | 0.91 | 187528 | Insulin-2 | IEDB |
| - | ASSPGQGGEQY | ASSQGGEQY | 0.91 | 58141 | Insulin-1,non-structural protein NS4b | IEDB |
| - | ASSRLGSAETLY | ASSPGLGSAETLY | 0.91 | 35060 | Insulin-2 | IEDB |
| - | ASSPGQGAEQF | ASSAGQGYEQY | 0.91 | 75802 | non-structural protein NS4b,Insulin-1,ORF3a protein [Severe acute respiratory syndrome coronavirus 2] | IEDB |
| - | ASSPGQGAEQF | ASSAGQGYEQY | 0.91 | 75802 | non-structural protein NS4b,Insulin-1,ORF3a protein [Severe acute respiratory syndrome coronavirus 2] | IEDB |
| - | ASSQGEGTEVF | ASSFSQGTEVF | 0.91 | 35034 | Insulin-2 | IEDB |
| - | ASGLQGYEQY | ASSLQGGYEQY | 0.91 | 35056 | Insulin-2 | IEDB |
| - | ASSSQHYEQY | ASSQGQYEQY | 0.91 | 56812 | Insulin-1 | IEDB |
| - | ASGNYAEQF | SADQGNYAEQF | 0.91 | 56897 | Insulin-1 | IEDB |
| - | ASGVQGYEQY | ASILSGYEQY | 0.91 | 56671 | Insulin-1 | IEDB |
| - | ASGVQGYEQY | ASILSGYEQY | 0.91 | 56671 | Insulin-1 | IEDB |
| - | ASGVQGYEQY | ASILSGYEQY | 0.91 | 56671 | Insulin-1 | IEDB |
| - | ASSPGQGTEQF | ASSPGQGNTEVF | 0.91 | 35061 | Insulin-2 | IEDB |
| - | ASSQQEGEQY | ASSQGQYEQY | 0.91 | 56812 | Insulin-1 | IEDB |
| - | ASSQIRNSPLY | ASSQDSNSPLY | 0.91 | 35146 | Insulin-2 | IEDB |
| - | ASSPDGSGNTLY | ASSQDTSGNTLY | 0.91 | 35150 | Insulin-2 | IEDB |
| - | ASSRGTGYEQY | TCSGTGYEQY | 0.91 | 35037 | Insulin-2 | IEDB |
| - | ASSLARGYEQY | ASSAGQGYEQY | 0.91 | 75802 | non-structural protein NS4b,Insulin-1,ORF3a protein [Severe acute respiratory syndrome coronavirus 2] | IEDB |
| - | ASSQGQGTEVF | ASSQGQGQDTQY | 0.91 | 35163 | Insulin-2 | IEDB |
| - | ASSAGTGGGNTLY | ASSAGSGNTLY | 0.91 | 35031 | Insulin-2 | IEDB |
| - | ASSQGASNERLF | ASSQISNERLF | 0.91 | 187558 | Insulin-2,Insulin-2 | IEDB |
| - | ASSSGQNTEVF | ASTGSQNTLY | 0.91 | 35075 | Insulin-2 | IEDB |
| - | ASSDAVNSPLY | ASSQDSNSPLY | 0.91 | 35146 | Insulin-2 | IEDB |
| - | ASSQAGGGSDYT | ASSIGTGGSDYT | 0.91 | 35117 | Insulin-2 | IEDB |
| - | ASSPGQNTEVF | ASSPGQGNTEVF | 0.91 | 35061 | Insulin-2 | IEDB |
| - | ASSPGQNTEVF | ASSPGQGNTEVF | 0.91 | 35061 | Insulin-2 | IEDB |
| - | ASSPGQNTEVF | ASSPGQGNTEVF | 0.91 | 35061 | Insulin-2 | IEDB |
| - | ASSLARGYEQY | ASILSGYEQY | 0.91 | 56671 | Insulin-1 | IEDB |
| - | ASSPGQGAGQF | ASSPGQGRAPL | 0.91 | 35134 | Insulin-2 | IEDB |
| - | ASSPGQGAEVF | ASSPGQGRAPL | 0.91 | 35134 | Insulin-2 | IEDB |
| - | ASSPGQGAEVF | ASSPGQGRAPL | 0.91 | 35134 | Insulin-2 | IEDB |
| - | ASSIVGVEQY | ASSQVGYEQY | 0.91 | 56696 | Insulin-1 | IEDB |
| - | ASSSGQGYEQY | ASSQGGEQY | 0.91 | 58141 | Insulin-1,non-structural protein NS4b | IEDB |
| - | ASTVGNSDYT | ASGDVTNSDYT | 0.91 | 35101 | Insulin-2 | IEDB |
| - | ASSQDLNSPLY | ASSQDINSDYT | 0.91 | 35141 | Insulin-2 | IEDB |
| - | ASSPGQGNERLF | ASNPGRANERLF | 0.91 | 56578 | Insulin-1 | IEDB |
| - | ASSMPVSNERLF | ASSQISNERLF | 0.91 | 187558 | Insulin-2 | IEDB |
| - | GARQGAGNTLY | ASSQQGSGNTLY | 0.91 | 35072 | Insulin-2 | IEDB |
| - | GARQGAGNTLY | ASSQQGSGNTLY | 0.91 | 35072 | Insulin-2 | IEDB |
| - | ASSTSAETLY | ASTGSQNTLY | 0.91 | 35075 | Insulin-2 | IEDB |
| - | ASGRQGYEQY | ASSAGQGYEQY | 0.91 | 75802 | non-structural protein NS4b,Insulin-1,ORF3a protein [Severe acute respiratory syndrome coronavirus 2] | IEDB |
| - | ASGRQGYEQY | ASSAGQGYEQY | 0.91 | 75802 | non-structural protein NS4b,Insulin-1,ORF3a protein [Severe acute respiratory syndrome coronavirus 2] | IEDB |
| - | ASSSGQGYEQY | ASSQGQYEQY | 0.91 | 56812 | Insulin-1 | IEDB |
| - | ASSPGQGAEQF | ASSPGQGRAPL | 0.91 | 35134 | Insulin-2 | IEDB |
| - | ASSPGQGAEQF | ASSPGQGRAPL | 0.91 | 35134 | Insulin-2 | IEDB |
| - | ASGLQGYEQY | ASSQVGYEQY | 0.91 | 56696 | Insulin-1 | IEDB |
| - | ASSSGQGYEQY | ASSLQGGYEQY | 0.91 | 35056 | Insulin-2 | IEDB |
| - | ASSQTGGLEQY | ASSLQGGYEQY | 0.91 | 35056 | Insulin-2 | IEDB |
| - | ASSPGQNTEVF | ASSPGTNERLF | 0.91 | 56745 | Insulin-1 | IEDB |
| - | ASSPGQNTEVF | ASSPGTNERLF | 0.91 | 56745 | Insulin-1 | IEDB |
| - | ASSPGQNTEVF | ASSPGTNERLF | 0.91 | 56745 | Insulin-1 | IEDB |
| - | AWSQKGLSNERLF | AWSLGGLSNERLF | 0.91 | 56877 | Insulin-1 | IEDB |
| - | ASSLRLSNERLF | ASSLRRSNTEVF | 0.91 | 35057 | Insulin-2 | IEDB |
| - | ASGRQGYEQY | ASSQVGYEQY | 0.91 | 56696 | Insulin-1 | IEDB |
| - | ASGRQGYEQY | ASSQVGYEQY | 0.91 | 56696 | Insulin-1 | IEDB |
| - | ASSFGQGIEQY | ASSFSQGTEVF | 0.91 | 35034 | Insulin-2 | IEDB |
| - | ASSPGQGTEVF | ASSFSQGTEVF | 0.91 | 35034 | Insulin-2 | IEDB |
| - | ASSPGQGTEVF | ASSFSQGTEVF | 0.91 | 35034 | Insulin-2 | IEDB |
| - | ASSPGQGTEVF | ASSFSQGTEVF | 0.91 | 35034 | Insulin-2 | IEDB |
| - | ASSPGQGAEVF | ASSPGLGAQY | 0.91 | 56740 | Insulin-1 | IEDB |
| - | ASSPGQGAEVF | ASSPGLGAQY | 0.91 | 56740 | Insulin-1 | IEDB |
| - | ASSQAGGGSDYT | ASSQDRGGGSDYT | 0.91 | 56777 | Insulin-1 | IEDB |
| - | ASSVLGGTQY | ASSILSGNTLY | 0.91 | 35118 | Insulin-2 | IEDB |
| - | ASSPDGSGNTLY | ASSQQGSGNTLY | 0.91 | 35072 | Insulin-2 | IEDB |
| - | ASSPGQGGEQY | ASSAGQGYEQY | 0.91 | 75802 | non-structural protein NS4b,Insulin-1,ORF3a protein [Severe acute respiratory syndrome coronavirus 2] | IEDB |
| - | ASSQGQGTEVF | ASSQGQTNQA | 0.91 | 56811 | Insulin-1 | IEDB |
| - | ASGLQGYEQY | ASSAGQGYEQY | 0.91 | 75802 | non-structural protein NS4b,Insulin-1,ORF3a protein [Severe acute respiratory syndrome coronavirus 2] | IEDB |
| - | ASSQIRNSPLY | ASSQISNERLF | 0.90 | 187558 | Insulin-2 | IEDB |
| - | ASSQGENTEVF | ASSQERTEVF | 0.90 | 35068 | Insulin-2 | IEDB |
| - | ASSQDTNSPLY | ASSRDTNSDYT | 0.90 | 35048 | Insulin-2 | IEDB |
| - | ASSQERGGEQY | ASSQGGEQY | 0.90 | 58141 | Insulin-1,non-structural protein NS4b | IEDB |
| - | ASSPGQGYEQY | ASSVGGQGYEQY | 0.90 | 35176 | Insulin-2 | IEDB |
| - | ASSLRLSNERLF | ASQISNERLF | 0.90 | 35029 | Insulin-2 | IEDB |
| - | ASSRLGSAETLY | ASSPRSSAETLY | 0.90 | 35136 | Insulin-2 | IEDB |
| - | TCSARQGNTGQLY | TCSADTGNTGQLY | 0.90 | 35083 | Insulin-2 | IEDB |
| - | ASSLAGQGAEQF | ASSAGQGYEQY | 0.90 | 75802 | non-structural protein NS4b,Insulin-1,ORF3a protein [Severe acute respiratory syndrome coronavirus 2] | IEDB |
| - | ASGTEGAEQF | ASSQGGEQY | 0.90 | 58141 | Insulin-1,non-structural protein NS4b | IEDB |
| - | ASSPGNTEVF | ASSPGTNERLF | 0.90 | 56745 | Insulin-1 | IEDB |
| - | ASSSGQGYEQY | ASSQVGYEQY | 0.90 | 56696 | Insulin-1 | IEDB |
| - | ASSLAGQGAEQF | ASSVGGQGYEQY | 0.90 | 35176 | Insulin-2 | IEDB |
| - | GARQGAGNTLY | ASSAGSGNTLY | 0.90 | 35031 | Insulin-2 | IEDB |
| - | GARQGAGNTLY | ASSAGSGNTLY | 0.90 | 35031 | Insulin-2 | IEDB |
| - | ASSPGQGAEQF | ASSPGLGAQY | 0.90 | 56740 | Insulin-1 | IEDB |
| - | ASSPGQGAEQF | ASSPGLGAQY | 0.90 | 56740 | Insulin-1 | IEDB |
| - | ASSLRGGEQY | ASSLQGGYEQY | 0.90 | 35056 | Insulin-2 | IEDB |
| - | ASRSGASSYEQY | ASSRDSSYEQY | 0.90 | 187578 | precore/core protein,Insulin-2 | IEDB |
| - | ASSLGGTSQNTLY | ASSLGSQNTLY | 0.90 | 187279 | pM45,Insulin-2 | IEDB |
| - | ASSPGQGTEQF | ASSAGQGYEQY | 0.90 | 75802 | non-structural protein NS4b,Insulin-1,ORF3a protein [Severe acute respiratory syndrome coronavirus 2] | IEDB |
| - | ASGETRTAAETLY | ASSQVTTSAETLY | 0.90 | 56698 | Insulin-1 | IEDB |
| - | ASSAGTGGGNTLY | ASTAGAGNTLY | 0.90 | 35179 | Insulin-2 | IEDB |
| - | ASSQDTNSPLY | ASSQDTNTGQLY | 0.90 | 193508 | Insulin,proSAAS [Mus musculus],proSAAS [Mus musculus] | IEDB |
| - | ASSDSAETLY | ASSPRSSAETLY | 0.90 | 35136 | Insulin-2 | IEDB |
| - | ASSPQGAGGTEVF | ASSQEGQGGTEVF | 0.90 | 35158 | Insulin-2 | IEDB |
| - | ASSPGNTEVF | ASSPGQGNTEVF | 0.90 | 35061 | Insulin-2 | IEDB |
| - | ASSRLGSAETLY | ASSMPGTAETLY | 0.90 | 35131 | Insulin-2 | IEDB |
| - | ASSQGQGTEVF | ASSQEGQGETLY | 0.90 | 56792 | Insulin-1 | IEDB |
| - | ASSQDMNSPLY | ASSQDINSDYT | 0.90 | 35141 | Insulin-2 | IEDB |
| - | ASTPGQGTEVF | ASSPGQGNTEVF | 0.90 | 35061 | Insulin-2 | IEDB |
| - | ASSQDAGGGYEQY | ASSDAGGRYEQY | 0.90 | 35112 | Insulin-2 | IEDB |
| - | ASSQDTNSPLY | ASSQDINSDYT | 0.90 | 35141 | Insulin-2 | IEDB |
| - | ASSPTTNSDYT | ASGDVTNSDYT | 0.90 | 35101 | Insulin-2 | IEDB |
| - | ASSGGVGNTLY | SAGGGQNTLY | 0.90 | 35193 | Insulin-2 | IEDB |
| - | TCSAGGQGTEVF | TCSAGGQGLDTQY | 0.90 | 35194 | Insulin-2 | IEDB |
| - | ASSRLGSAETLY | ASSLGSQNTLY | 0.90 | 187279 | pM45,Insulin-2 | IEDB |
| - | ASSQDPAGNTLY | ASSPDSSGNTLY | 0.90 | 56738 | Insulin-1 | IEDB |
| - | ASGDAGGGNTLY | ASSAGSGNTLY | 0.90 | 35031 | Insulin-2 | IEDB |
| - | ASSTGTGGDEQY | ASSIGTGGSDYT | 0.90 | 35117 | Insulin-2 | IEDB |
| - | ASSTGTGGDEQY | ASSIGTGGSDYT | 0.90 | 35117 | Insulin-2 | IEDB |
| - | ASRNWGSYEQY | ASSQDWGSYEQY | 0.90 | 187540 | Insulin-2 | IEDB |
| - | ASSQPGQGYEQY | ASSVGGQGYEQY | 0.90 | 35176 | Insulin-2 | IEDB |
| - | ASSPGQGTEVF | ASSPGQGRAPL | 0.90 | 35134 | Insulin-2 | IEDB |
| - | ASSPGQGTEVF | ASSPGQGRAPL | 0.90 | 35134 | Insulin-2 | IEDB |
| - | ASSPGQGTEVF | ASSPGQGRAPL | 0.90 | 35134 | Insulin-2 | IEDB |
| - | ASSPGNSPLY | ASSPRESPLY | 0.90 | 35063 | Insulin-2 | IEDB |
| - | ASSPGNSPLY | ASSPRESPLY | 0.90 | 35063 | Insulin-2 | IEDB |
| - | ASSIGQGYEQY | ASILSGYEQY | 0.90 | 56671 | Insulin-1 | IEDB |
| - | ASSQGEGTEVF | ASSQEGQGETLY | 0.90 | 56792 | Insulin-1 | IEDB |
| - | ASKRTGANERLF | ASSQEKGANERLF | 0.90 | 56795 | Insulin-1 | IEDB |
| - | GARVGNSDYT | GARVGADSDYT | 0.90 | 35188 | Insulin-2 | IEDB |
| - | ASGVQGYEQY | ASSLQGGYEQY | 0.90 | 35056 | Insulin-2 | IEDB |
| - | ASGVQGYEQY | ASSLQGGYEQY | 0.90 | 35056 | Insulin-2 | IEDB |
| - | ASGVQGYEQY | ASSLQGGYEQY | 0.90 | 35056 | Insulin-2 | IEDB |
| - | ASSQGASNERLF | ASGEGGTNERLF | 0.90 | 35102 | Insulin-2 | IEDB |
| - | ASSLVQRDTQY | ASSLQGQDTQY | 0.90 | 56717 | Insulin-1 | IEDB |
| - | ASSRVGQGGEQY | ASSQAGGRGEQY | 0.90 | 35137 | Insulin-2 | IEDB |
| - | ASSQGQNTEVF | ASSFSQGTEVF | 0.90 | 35034 | Insulin-2 | IEDB |
| - | ASSQGQNTEVF | ASSFSQGTEVF | 0.90 | 35034 | Insulin-2 | IEDB |
| - | ASSPGQGAGQF | ASSPGLGAQY | 0.90 | 56740 | Insulin-1 | IEDB |
| - | ASSGGGSNERLF | GAGGGLNERLF | 0.90 | 35185 | Insulin-2 | IEDB |
| - | ASSSGTGVEQF | ASSAGSGNTLY | 0.90 | 35031 | Insulin-2 | IEDB |
| - | ASSRTDSNERLF | ASSQISNERLF | 0.90 | 187558 | Insulin-2 | IEDB |
| - | ASSIGQGYEQY | ASSQVGYEQY | 0.90 | 56696 | Insulin-1 | IEDB |
| - | ASGDAGGGNTLY | ASTAGAGNTLY | 0.90 | 35179 | Insulin-2 | IEDB |
| - | ASSQTGGLEQY | ASSQGGEQY | 0.90 | 58141 | Insulin-1,non-structural protein NS4b | IEDB |
| - | ASSSGQNTEVF | ASSQGQTNQA | 0.90 | 56811 | Insulin-1 | IEDB |
| - | ASSPGQGAEVF | ASSPGQGNTEVF | 0.90 | 35061 | Insulin-2 | IEDB |
| - | ASSPGQGAEVF | ASSPGQGNTEVF | 0.90 | 35061 | Insulin-2 | IEDB |

Islet-derived CDR3-beta sequences were queried using the IEDB TCRMatch Tool with a threshold of 0.90 for antigen specificity prediction based on sequence similarity.
